# Managing Forests for Biodiversity Conservation and Climate Change Mitigation

**DOI:** 10.1021/acs.est.3c07163

**Published:** 2024-05-14

**Authors:** Cindy G. Azuero-Pedraza, Pekka Lauri, Andrey Lessa Derci Augustynczik, Valerie M. Thomas

**Affiliations:** † H. Milton Stewart School of Industrial and Systems Engineering, Georgia Institute of Technology, Atlanta, Georgia 30332, United States; ‡ International Institute for Applied Systems Analysis (IIASA), Schlossplatz 1, Laxenburg A-2361, Austria; § CMCC Foundation—Euro-Mediterranean Center on Climate Change, Via Marco Biagi 5, Lecce 73100, Italy; ∥ RFF-CMCC European Institute on Economics and the Environment, Via Bergognone 34, Milan 20144, Italy; ⊥ School of Public Policy, Georgia Institute of Technology, Atlanta, Georgia 30332, United States

**Keywords:** biodiversity, forest management, partial equilibrium, bioenergy, woody biomass, climate change mitigation, linear programming

## Abstract

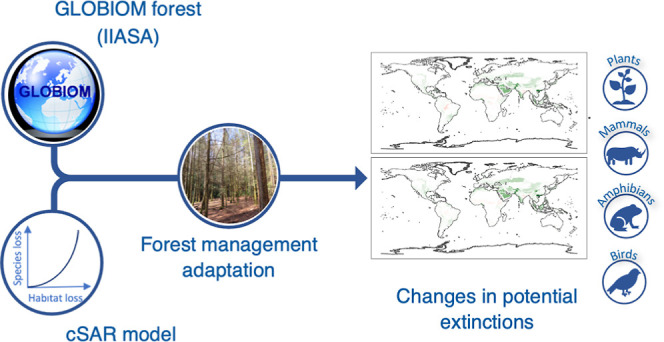

We include biodiversity impacts in forest management decision making by incorporating the countryside species area relationship model into the partial equilibrium model GLOBIOM-Forest. We tested three forest management intensities (low, medium, and high) and limited biodiversity loss via an additional constraint on regional species loss. We analyzed two scenarios for climate change mitigation. RCP1.9, the higher mitigation scenario, has more biodiversity loss than the reference RCP7.0, suggesting a trade-off between climate change mitigation, with increased bioenergy use, and biodiversity conservation in forests. This trade-off can be alleviated with biodiversity-conscious forest management by (1) shifting biomass production destined to bioenergy from forests to energy crops, (2) increasing areas under unmanaged secondary forest, (3) reducing forest management intensity, and (4) reallocating biomass production between and within regions. With these mechanisms, it is possible to reduce potential global biodiversity loss by 10% with minor changes in economic outcomes. The global aggregated reduction in biodiversity impacts does not imply that biodiversity impacts are reduced in each ecoregion. We exemplify how to connect an ecologic and an economic model to identify trade-offs, challenges, and possibilities for improved decisions. We acknowledge the limitations of this approach, especially of measuring and projecting biodiversity loss.

## Abstract 2

We incorporate biodiversity into forest management decisions to show how biodiversity-conscious decision making can alleviate the trade-off between biodiversity conservation and climate change mitigation with high biomass requirements.

## 1 Introduction

Current and future land use affects biodiversity.^1,2^ suggest that future global land use change scenarios, especially those with strong climate change mitigation using bioenergy, will have negative impacts on biodiversity. Similarly, Heck et al.^3^ indicate how bioenergy with carbon capture and storage (BECCS) potentially required for climate change mitigation surpasses the planetary boundary of biosphere integrity, measured by the Biodiversity Intactness Index. Hof et al.^4^ find that the impact of bioenergy cropland expansion on global terrestrial species richness could offset the positive effects of prevented climate change.^4^ In the context of forests, land use change usually refers to deforestation and loss of wilderness areas.

A less explored path is the potential of forest management decisions to mitigate the trade-offs between climate protection and biodiversity conservation. Some authors have mentioned that management intensity affects biodiversity impacts^5−9^ and that more sustainable forest management can reduce negative impacts on biodiversity.

However, current models used to represent and assess forest management decisions typically do not incorporate biodiversity impacts, e.g., the Global Timber Model^10−12^ and GLOBIOM-Forest.^13,14^ These partial equilibrium economic models are used in isolation to understand the effects of several scenarios and policies on forests and forest markets and are also used in combination with other land use models or integrated assessment models (IAMs) to represent forestry sector decisions within a larger interconnected system.

These partial equilibrium models incorporate forest product markets where consumers and producers interact with prices, determining the quantities produced. Since these partial equilibrium models reflect only market goods, they ignore nonmarket goods, such as biodiversity, ecosystem services, and other externalities associated with the forest industry. This gap implies that the forest management decisions made using these models are missing the opportunity to improve biodiversity outcomes.

By incorporating biodiversity into forest management decision making, we can identify ways through which the forestry sector can reduce its impacts on biodiversity and alleviate the potential trade-off with climate change mitigation strategies that use high levels of biomass. The objectives of this study are (1) to propose a way to incorporate biodiversity into forest management decision making by integrating an economic and an ecologic model, (2) to identify how biomass production and the forest industry are affected when constraining biodiversity impacts of biomass production, and (3) to show mechanisms through which biomass production could reduce its impacts on biodiversity while still producing the biomass demands for bioenergy.

The paper is structured as follows. The first section describes the economic (GLOBIOM-Forest) and ecologic (countryside species area relationship model—cSAR) models used. The second section explains how the cSAR model is integrated into GLOBIOM-Forest. In the third section, the results are shown for two scenarios that correspond to the combination between two climate change scenarios and a constraint on biodiversity loss.

## 2 Methodology

An existing ecological model, the cSAR model, is incorporated into an existing economic model, GLOBIOM-Forest. The following sections describe the economic model, the cSAR model and how it was adapted to forest management decisions, the modifications required to integrate both models, and the scenarios.

### 2.1 GLOBIOM-Forest

The IIASA Global Biosphere Management Model (GLOBIOM)^45,46^ has been developed since 2000 for integrated assessment of climate change mitigation policies involving land use, such as biofuels (https://iiasa.github.io/GLOBIOM/). It is a bottom-up partial equilibrium model in which total economic surplus, typically used measure to represent social welfare in economics, is maximized. As such, the model represents economically optimal behavior subject to the model constraints. GLOBIOM-Forest is a version of GLOBIOM with greater forest detail^13,14^. Supply is represented on a spatially explicit basis for a grid of 200 km × 200 km (), while demand and bilateral trade are represented on a regional basis (with up to 58 economic regions—Table S4). Supply is then aggregated to the economic regions to guarantee that supply equals demand. GLOBIOM-Forest includes a representation of both forestry and the forest industry, considering harvested products, byproducts, intermediate products, and final products. Forests are categorized as unmanaged forests and managed forests: *unmanaged forests* are not harvested and include the primary and secondary forest types: primary—naturally regenerated forests of native tree species where there are no clearly visible indications of human activity and the ecological processes are not significantly disturbed and secondary—a forest or woodland area which has regrown after a major disturbance but is not yet at the end point of succession and managed forests. *Managed forests* are harvested to obtain woody biomass, with low, medium, or high-intensity management. These are distinguished from *energy crops* represented via short rotation plantations (SRP), which are not considered to be forest land but are located in agricultural land or grassland. The model is used to represent two types of supply decisions: on forest management, and on land use change for energy crops. These decisions are made on a spatially explicit basis considering the grid and the country level. On the one hand, there is forest management, which takes place in forestland. Forestland area remains constant in the model since afforestation and reforestation are not considered in the version used. In this constant forest area, the forest management decisions are made. The types of forest management correspond to set *F* = {primary forest, secondary forest, low-intensity management, medium-intensity management, high-intensity management} (see Table S3 for definitions of each forest management type).

On the other hand, there is land use change. The representation of land use change in GLOBIOM-Forest is simplified. It does not consider all land use types, nor all possible land use transformations. It only represents the transformation from suitable agricultural land or suitable grasslands to SRP. In this way, SRP are not located in forestland, and under the model definitions are not considered forests, but energy crops.

The connection between the two decisions is through bioenergy demand for biomass. Exogeneous bioenergy demand for biomass (for nontraditional use) can be satisfied with both biomass produced in forest land under the different management types or biomass being produced in SRP. In contrast, endogenous biomass demand for wood products can only be satisfied with biomass being produced in forestland.

GLOBIOM-Forest is solved as a linear programming model recursively with 10 year intervals for the years 2000–2100 with a calibration period between 2000 and 2020. Each year the economic surplus (see eq S14) is maximized. For more information on GLOBIOM-Forest, see the Supporting Information, or refs (13 and 14).

### 2.2 Biodiversity Model Assessment

In this analysis, biodiversity impacts are estimated as a response to habitat loss driven by changes in forest management and energy crops. This includes (1) changes from primary and secondary forests to each of the three levels of intensity of managed forests, (2) changes in intensity in already managed forests, (3) changes from managed forests to secondary forests, and (4) changes from suitable agricultural lands or grasslands to SRP. Only the species-level dimension of biodiversity is included. We implement the cSAR model^15,16^. The indicator estimated by the model (*Slost*_*g*,*l*_^Regional^) is the potential regional species loss or species extirpation for taxon *g* in ecoregion *l*. This indicator can be interpreted as the number of species committed to extinction due to habitat loss, in comparison to an original scenario, in each World Wildlife Fund (WWF) ecoregion. An ecoregion is a biogeographical characterization made by the WWF of land that shares a large majority of species, dynamics, and environmental conditions^17^. Note that the disappearance of a species in an ecoregion does not imply the disappearance of the species on a global basis; therefore this indicator does not correspond to global extinctions. The cSAR model is an empirically derived relationship between areas of habitat lost and species lost, with a characteristic scaling factor *z*_*l*_. The model^15,16,18^ used here, eq 1, follows the presentation by Chaudhary and colleagues^19^, adapted to forest management and energy crops consistently with GLOBIOM-Forest modeling assumptions. It is based on refs (7, 19 and 20). For more details on the model integration and its limitations, see ref (21).
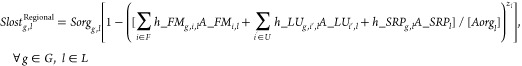
1

In eq 1, *G* is the set of taxonomic groups and *L* is the set of ecoregions. Forest management types *i* in GLOBIOM-Forest correspond to the set *F* = {primary forest, secondary forest, low-intensity management, medium-intensity management, high-intensity management}. A description of each forest management type can be seen in Supporting Information Table S3. Land use types *i*′ with suitable areas for SRP correspond to the set *U* = {agricultural land, grassland, other natural land}.

The model is based on the proportion of the areas available for species in a reference scenario versus the area available for species in the new future scenario. The former will correspond to the denominator of the fraction inside the parentheses and the latter will correspond to the numerator. *Aorg*_*l*_ and *Sorg*_*g*,*l*_ are the amount of natural habitat and the number of species of taxon *g* in the reference scenario in ecoregion *l*, respectively. *A*_*FM*_*i*,*l*_, *A*_*LU*_*i*′,*l*_, and *A*_*SRP*_*l*_ are the amount of area devoted to each forest management type *i*, each land use type *i*′ and SRP, respectively, on each ecoregion *l* in the future scenario. *h*_*FM*_*g*,*i*,*l*_, *h*_*LU*_*g*,*i*′,*l*,_ and *h*_*SRP*_*g*,*l*_ represent the affinity of the taxonomic group *g* for forest management type *i*, for land use type *i*′ and SRP, respectively, on ecoregion *l*. *z*_*l*_ is a constant from the classic SAR model for ecoregion *l* that reflects how rapidly species are lost due to habitat loss.

A typical assumption when assessing biodiversity changes is to use this reference scenario as a pristine scenario without human intervention^22^. We followed this assumption which implies *Aorg*_*g*,*l*_ = *TotalArea*_*l*_. In this adaptation of the cSAR model, *TotalArea*_*l*_ corresponds to the total forest area plus the area from agricultural land, grasslands, and other natural land that is suitable for SRP. This is not the total area of ecoregion *l* as is usually considered in previous uses of the model. The following relation remains ∑_*i*_*A*_*FM*_*i*,*l*_ + ∑_*i*′_*A*_*LU*_*i*′,*l*_ + *A*_*SRP*_*l*_ = *TotalArea*_*l*_ for each ecoregion *l*. The affinities, *h*_*FM*, *h*_*LU* and *h*_*SRP* ∈ [0, 1], can be interpreted as the proportion of the area under each management type *i*, land use type *i*′ or SRP, respectively, that can be used by the taxon *g* in each ecoregion *l*(15,16). In this way, *Slost*_*g*,*l*_^Regional^ corresponds to the potential species loss or extirpation in each ecoregion due to the decrease of habitat from *Aorg*_*l*_ to ∑_*i*∈*F*_*h*_*FM*_*g*,*i*,*l*_*A*_*FM*_*i*,*l*_ + ∑_*i*′∈*U*_*h*_*LU*_*g*,*i*′,*l*_*A*_*LU*_*i*′,*l*_ + *h*_*SRP*_*g*,*l*_*A*_*SRP*_*l*_.

There are significant limitations of the cSAR model; among which is the fact that it only represents the species-level dimension of biodiversity. More limitations are acknowledged in the Discussion section. Other models of biodiversity could be considered; we discuss them elsewhere^21^.

#### 2.2.1 Data for the Biodiversity Model

The affinity factors *h*_*FM*_*g*,*i*,*l*_ are derived from ref (7). Let *CMng* be the set of the ten forest management types included in ref (7) and *Con* be the set of continents. Then, the mean response rate *R*_*i*″,*c*,*g*_ with *i*″ ∈ *CMng*, *c* ∈ *Con* and *g* ∈ *G* is taken from Supporting Information of ref (7). With these response rates and a *z*_*l*_ = 0.344, ∀ *l* ∈ *L*(23), *h*_*i*″,*c*,*g*_ is calculated as in eq 2.
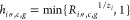
2

The minimum in eq 2 ensures that affinities are not greater than 1, at which value species can use all of the modified habitat. These affinities *h*_*i*″,*c*,*g*_ are then transformed to obtain the affinities *h*_*FM*_*g*,*i*,*l*_ required for the model integration. The mapping between Chaudhary and colleagues’s^7^ management types and GLOBIOM-Forest management types (see Table S1) is used. The affinities of an ecoregion *l* correspond to the affinity of the continent *c* to which they belong. If an ecoregion has territory over more than one continent, a weighted average using the area in each continent is used.

The affinity factors *h*_*LU*_*g*,*i*′,*l*_ for suitable land for SRP and those for SRP, i.e., *h*_*SRP*_*g*,*l*_ are derived from ref (19) using the information in their Supporting Information on *CFloc* and indicated procedure. The mapping between^19^ land use types and those in set *U* from GLOBIOM-Forest (see Table S2) is used.

In this implementation, *Aorg*_*l*_ corresponds to the sum of forest area and the area suitable for SRP in the ecoregion *l*. These areas were calculated using data from GLOBIOM-Forest and a mapping between GLOBIOM-Forest spatial units *s* and the ecoregions *l*. The mapping corresponds to the intersection between the ecoregions map and the GLOBIOM-Forest spatial units (on a 200 km × 200 km basis) map done in ArcGIS. This intersection map provides the weights *mW*_*s*,*l*_ of spatial unit *s* in ecoregion *l*. Accordingly
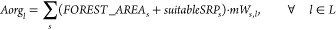
3

In eq 3, *FOREST*_*AREA*_*s*_ is the forest area in the spatial unit *s*, whereas *suitableSRP*_*s*_ is the total area suitable for SRP considered on GLOBIOM-Forest, i.e., *suitableSRP*_*s*_ = ∑_*i*′_*SRP*_*DATA*_*s*,*i*′_ where *SRP*_*DATA*_*s*,*i*′_ is the suitable area for SRP from each land use type *i*′ ∈ *U* in that spatial unit *s*. The area under each forest management type *i* in each ecoregion *l*, *A*_*ForMng*_*i*,*l*_, the area under each land use type *i*′ in each ecoregion *l*, *A*_*LU*_*i*′,*l*_ and the area under SRP, *A*_*SRP*_*l*_, were estimated similarly according to Supporting Information eqs S1–S3, respectively. These equations connect the biodiversity model and the forestry model.

Data on *Sorg*_*g*,*l*_^a^ comes from ref (20). Finally, *z*_*l*_ = 0.344, ∀ *l* ∈ *L*, corresponding to the mean value for forests according to ref (23).

Only amphibians, birds, mammals, and plants are considered in the set of taxa due to data limitations.

For more information on how data were constructed, R codes are available at https://github.com/cga203/cSAR-GLOBIOM_Forest.

### 2.3 Integration of the Models

There are two main components of the integration between the cSAR model and GLOBIOM-Forest: the data mappings and the methodology to incorporate biodiversity into forest management decisions. Data mappings are necessary for the spatial units, the management types, and the time periods (see Supporting Information Section S2 for a detailed description).

To incorporate biodiversity into forest management decisions, the cSAR model was connected to both the decision variable in GLOBIOM-Forest that represents the area of forest that will be harvested in each spatial unit under each forest management type and the variable that represents land use change from suitable land to SRP. A constraint was added to limit the amount of biodiversity impact that results from the management decisions. The biodiversity impact, as estimated by the cSAR model (*Slost*_*g*,*l*_^Regional^), is aggregated among all ecoregions and is limited for each taxon (see eq 4). In this constraint, ∑_*l*∈*L*_*Slost*_*g*,*l*_^Regional^ has a special interpretation. It reflects the sum of the number of regional species committed to extinction (or extirpations), which differs from the number of global species committed to extinction. A species could disappear in one ecoregion but remain present in another. *Bmax*_*g*_ represents the upper bound on biodiversity loss, for each taxon, as defined in the Supporting Information. This follows the methodology proposed by ref (21) using a piecewise linear approximation to represent the cSAR model. For more details on the specific integration for GLOBIOM-Forest, see the Supporting Information.
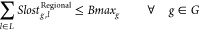
4

It is important to note that forest management decisions and land use change decisions for SRP are made on GLOBIOM-Forest’s spatial units (grid intersection with countries), whereas biodiversity impacts are estimated on an ecoregion level. GLOBIOM-Forest’s spatial units are contained into countries, which are contained into economic regions. This allows the aggregation of the supply results for comparison and analysis of biomass production at the level of economic regions. Ecoregions are not contained in countries; one ecoregion can cover more than one country and one country can have more than one ecoregion.

### 2.4 Analyzed Scenarios

The model was run for eight scenarios, shown in Table 1, corresponding to the combination of four biodiversity loss thresholds (see Supporting Information Section S5) and two climate change mitigation scenarios. An increase in the percentage of biodiversity loss reduction represents a tighter constraint. This means that the scenario with a 40% reduction in biodiversity loss must have a smaller feasible solution space compared to that of the 10% reduction scenario.

**Table 1:** Eight Scenarios Analyzed Corresponding to the Combination of Four Biodiversity Thresholds and Two Mitigation Scenarios

	Biodiversity (*Bmax*)			
Mitigation scenario	SSP2-RCP1.9	SSP2-RCP1.9	SSP2-RCP1.9	SSP2-RCP1.9
	10% reduction w.r.t. baseline	20% reduction w.r.t. baseline	30% reduction w.r.t. baseline	40% reduction w.r.t. baseline
			
	SSP2-RCPref	SSP2-RCPref	SSP2-RCPref	SSP2-RCPref
	10% reduction w.r.t. baseline	20% reduction w.r.t. baseline	30% reduction w.r.t. baseline	40% reduction w.r.t. baseline

The climate change mitigation scenarios chosen are RCPref^24^ (which is close to RCP7.0) and RCP1.9^25^, used in conjunction with SSP2 shared socio-economic pathway^26^, that characterizes overall socio-economic development. This follows the current framework^27^ for the study of climate-related scenario outcomes. RCPref is a no-mitigation scenario, with high greenhouse gas emissions, where harvest volumes do not increase much over time. This corresponds to business as usual. In contrast, RCP1.9, which is consistent with the 1.5 °C target, corresponds to a high mitigation overshoot scenario that increases harvest volumes after 2020. In it, there is a significant increase in bioenergy demand, which for GLOBIOM-Forest is exogenously defined according to bioenergy demand from the MESSAGE IAM^24^. Bioenergy demands from MESSAGE are the same for both scenarios until 2020 with a level of 57 EJ in 2020. Then, RCP1.9 increases significantly each year reaching 225.5 EJ by 2100, while under RCPref bioenergy demand grows slowly with a value of 65.7 EJ by 2100. Differences in bioenergy demand from MESSAGE are shown in Supporting Information Figure S1. Accordingly, using RCPref and RCP1.9, we have two extremes on the spectrum of climate change mitigation pathways.

The SSP Public Database^26^ indicates that these RCP1.9 bioenergy demands correspond to 28% of the total primary energy requirements in 2100. In contrast, the bioenergy demands in 2100 for RCPref correspond to 5% of the total primary energy requirements.

## 3 Results

### 3.1 Attainability of Scenarios

We find that not all percentage reductions in biodiversity loss are attainable. Scenarios with 20, 30, and 40% reductions are infeasible under current assumptions in GLOBIOM-Forest and the integration of the cSAR biodiversity model. These infeasibilities occur in both mitigation scenarios. Not finding a feasible solution for these scenarios implies that the mechanisms available for the model to reduce biodiversity impacts and increase woody biomass production at the same time are insufficient to attain both the desired improvement in biodiversity outcomes and the increased biomass production for future years.

### 3.2 Outcomes

#### 3.2.1 Biodiversity Impacts

**Figure 1:**
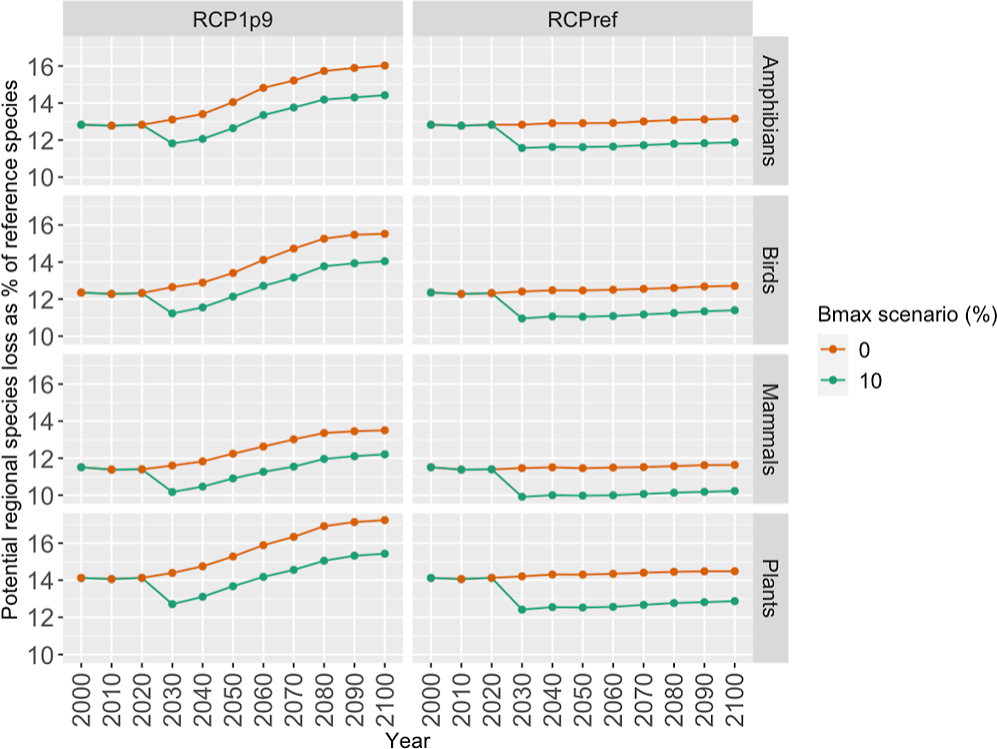
Projected total biodiversity impact, per taxon, for scenarios that limit biodiversity loss, between 2000 and 2100. The *y*-axis shows the total sum of extirpations between ecoregions as a percentage of the number of reference species included in the model, i.e., ∑_*l*∈*L*_*Slost*_*g*,*l*_^Regional^/∑_*l*∈*L*_*Sorg*_*g*,*l*_ ∀ *g* ∈ *G*.

Figure 1 shows the projected total biodiversity impact, per taxon, for the scenario imposing biodiversity limits under the two climate change mitigation scenarios. The total biodiversity impact is presented as the total regional species loss (or the total sum of extirpations) as a % of the number of species in the reference scenario *Sorg*_*g*,*l*_ by taxa, i.e., ∑_*l*∈*L*_*Slost*_*g*,*l*_^Regional^/∑_*l*∈*L*_*Sorg*_*g*,*l*_ ∀ *g* ∈ *G*. The figure shows that there is a potential trade-off between climate change mitigation, with increased bioenergy use, and biodiversity conservation. We find a higher biodiversity impact, from habitat loss, for the high mitigation scenario (RCP1.9), in comparison to the no-mitigation scenario (RCPref). This holds for all years in the pathway. The reasons are the following: (1) RCP1.9 has higher biomass demand (see Figure S1) that results in greater land requirements for managed forests (see Figure S7a,b, for 2030 and 2100, respectively) and higher management intensity (see Figures S6 and 4 for 2030 and Figures S8 and S9 for 2100), (2) no afforestation or deforestation are included, so the effects of potentially increased forest area, under RCP1.9, on biodiversity are not considered, and (3) estimated impacts on biodiversity are only for habitat loss. Under a constant forest area, there is a trade-off between biodiversity impacts due to habitat loss and woody biomass production for bioenergy.

For about the same levels of economic outcomes (see the section on Economic Outcomes), biodiversity impacts can be reduced by 10%, for all taxa on a global basis, by changing forest management decisions. This means that by incorporating biodiversity impacts into forest management decision making we can identify better solutions for both biodiversity conservation and biomass production from forests. With these reductions in biodiversity loss, for the high mitigation scenario (RCP1.9), we can keep the number of species committed to extinction closer to current levels. For the no-mitigation scenario (RCPref), we could even reverse the trend of increased biodiversity loss (see Figure 1). Notice how introducing the constraint per taxa (see eq 4) imposes the same proportional reductions in biodiversity loss on all taxa.

Since the constraint on biodiversity (eq 4) is defined on a global basis (aggregating over all ecoregions), it guarantees a global reduction of potential species loss for each taxon. This global constraint allows the model to shift biodiversity impacts between ecoregions. This results in some combinations of ecoregions and taxa with more species loss and others with less species loss when the constraint is introduced. Supporting Information Figure S3 shows an example of regional differences in potential mammal species loss among ecoregions. In it, for the biodiversity constraint and climate mitigation scenario, 10% loss reduction and RCP1.9, the model estimates less mammal species loss in ecoregions in the United States, Central America, Colombia, southeast Brazil, Uruguay, northern Africa, southern Africa, Tanzania, Mozambique, southern India, Pakistan, China, and Kazakhstan among others. On the other hand, there is more potential species loss in northern Brazil, western Africa, Somalia, Ethiopia, and east India. These regional differences are associated with the reallocation of forest management types and biomass production across and between regions. These regional results are not meant to be prescriptive, but to show how, even when improving results on global-level biodiversity outcomes, results may not improve in all ecoregions. This is further discussed later.

Satisfying the biodiversity loss constraint is harder for amphibians than for the other taxa. Table 2 shows the dual variables (shadow prices) associated with the biodiversity constraints for each taxon, each climate change mitigation scenario, and the 10% reduction scenario for biodiversity loss. Dual variables are presented for both 2030 and 2100. These values can be interpreted as the cost for the model to satisfy the constraint. We can see that for all scenarios presented in the table, it is harder to satisfy the constraint for amphibians than for other taxa. Indeed the shadow prices suggest that once changes in forest management and SRP are made to protect amphibians, no additional changes are required to satisfy the constraint for other taxa. This may be associated with the fact that amphibians’ reference species richness is concentrated in fewer ecoregions than other taxa (see Figure S13), limiting the flexibility of the model to reallocate production when enforcing a constraint for each taxon. Furthermore, as discussed in the Sensitivity section, the behavior of shadow prices for each taxon varies depending on the way in which the biodiversity constraint is introduced.

**Table 2:** Shadow Price of Restricting Biodiversity Loss for Years 2030 and 2100 Under the 10% Biodiversity Loss Reduction Scenario and for Each Climate Change Mitigation Scenario^a^

	2030		2100	
Taxa	RCP1.9	RCPref	RCP1.9	RCPref
Amphibians	318,564	640,329	12,179	20,993
Birds	0	0	0	0
Mammals	0	0	0	0
Plants	0	0	0	0

a The shadow price or dual variable associated with constraint 4 shows how would the total global economic surplus change if the biodiversity constraint was relaxed by 1 unit.

The shadow prices can also be interpreted as the opportunity cost, in terms of economic surplus, of not committing one species of a specific taxon to extinction. For example, Table 2 shows that if we allow 1 more amphibian species to be committed to extinction in 2100 (i.e., increasing *Bmax*_amphibians_ by 1), the global total economic surplus of the forest sector would increase by 12,179 (2020 USD) under the high mitigation scenario. For comparison, the total global economic surplus ranges around 19 billion (2020 USD) for 2100.

Table 2 also shows it is harder for the model to satisfy the constraints in 2030, in comparison to 2100. This is because the biodiversity constraint was introduced in 2030. 2030 is the first period in which the model has to reallocate production to consider biodiversity loss. Similarly, the no-mitigation scenario (RCPref) has higher shadow prices than the high mitigation scenario (RCP1.9). This may be due to the fact that even when both reduction scenarios correspond to a 10% with respect to the baseline (without constraining biodiversity loss), the baseline value for biodiversity loss is higher for RCP1.9 than for RCPref. Then, in absolute values, the constraint is tighter for the RCPref scenario.

#### 3.2.2 Economic Outcomes

Economic outcomes will be measured by the production of biomass in forests. This can be represented by the harvest volumes of roundwood (RW), in million *m*^3^, which aggregates the production of sawnlogs (SW_Biomass), pulplogs (PW_Biomass), other industrial RW (OW_Biomass), and fuelwood (FW_Biomass). With the introduction of the biodiversity constraint, the largest reduction in global RW production in forests, across scenarios, is 8.41%, passing from 4,871 million *m*^3^ in the baseline scenario to 4,461 million *m*^3^ under RCP1.9 and the 10% reduction scenario for 2030. Under this scenario, 250 amphibians, 3,058 birds, 1,045 mammals, and 26,371 plant species are no longer committed to extinction regionally (extirpated) because of habitat loss. Adding over all taxa, there is an overall reduction in regional species loss of 30,724 species. This can be interpreted as an average opportunity cost of 13,328 *m*^3^/species.

Total global economic surplus, the objective function of the optimization problem, has even smaller changes. The largest reduction on a global basis is 0.24% passing from 14,663 million (2020 USD) to 14,628 million (2020 USD) for the RCPref and 10% reduction scenario in 2030. More details about the small changes in economic outcomes are in the Supporting Information sections on biomass production and economic surplus.

**Figure 2:**
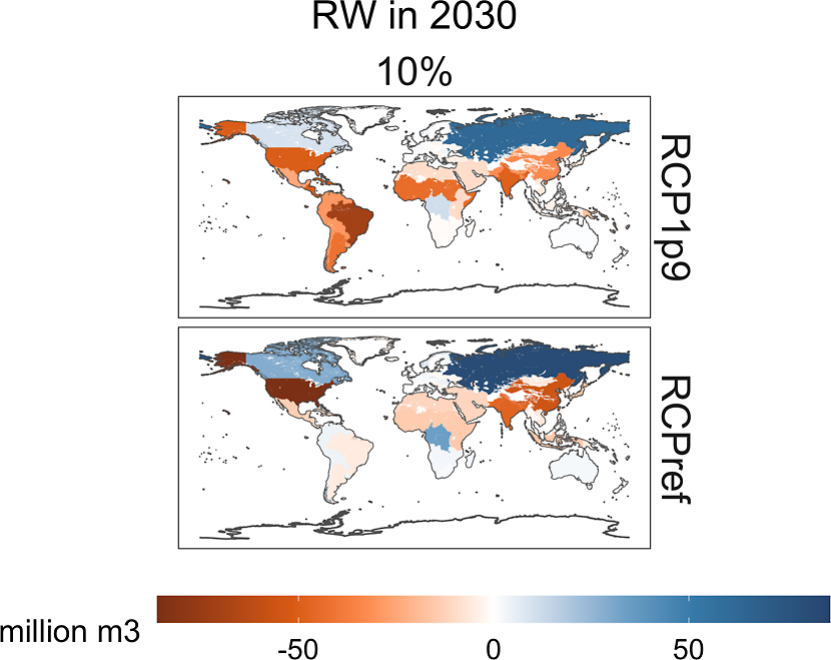
Regional differences between the eight scenarios assessed and the baseline (without the inclusion of biodiversity), for RW biomass production in forests, in 2030.

Economic impacts differ between the global and the regional scale. As shown in Figure 2, the estimated reduction in biomass production for 2030 corresponds to the net effect of economic regions with increased production (in blue) and regions with decreased production (in orange). For example, across scenarios, the economic regions of the Former USSR, Canada, and Congo basin region show increased production levels whereas India, China, United States, Argentina, and Brazil show decreased production levels. The economic regions with the largest relative changes to their baseline production are Argentina region with a 71% reduction (RCP1.9, 10% scenario) and Slovenia with a 57% increase (RCPref, 10% scenario) in 2070.

### 3.3 How Does the Model Achieve These Outcomes?

#### 3.3.1 Energy Crops and Land Use Change

**Figure 3:**
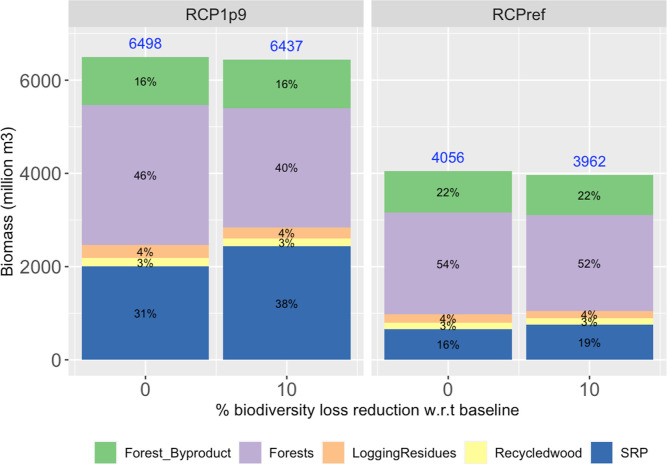
Sources of biomass for energy use for increased degrees of biodiversity loss reduction, on a global basis, for 2030. In blue numbers, the total biomass produced for energy use under each scenario. Energy use includes both traditional and nontraditional energy use. Nontraditional corresponds to the exogeneously defined amounts from RCP scenarios.

For biomass for energy use, there is a shift in production from forests to energy crops (SRP). The major change is for RCP1.9, with a shift of about 7% from forest to SRP contribution in 2030 (see Figure 3). Energy use includes both traditional and nontraditional energy use. Nontraditional energy use demanded quantities are exogenously defined from RCP scenarios, whereas traditional biomass demanded quantities respond endogenously to changes in biomass production. Traditional biomass demanded quantities are small compared to nontraditional biomass ones. In the model, biomass for nontraditional energy use can be supplied with both biomass coming from forests and biomass from SRP. When introducing the biodiversity constraint, two effects happen in all scenarios. First, there is a small reduction in the total amount of biomass produced for energy use because of the endogenous response to the demand for traditional use. This can be seen in the blue numbers in Figure 3. Second, due to the exogenous demand for nontraditional energy use and the fact that nontraditional biomass demand drives the demand for energy use, there is a shift from producing biomass in forests to producing biomass in SRP.

To increase biomass production in SRP, land use change is required. Since in the model SRP yields remain constant through time, increases in biomass production in SRP imply increased requirement for land. Land for SRP can be obtained from suitable agricultural land or suitable grassland. For the shift of 7% in production from forests to SRP (for RCP1.9, 10% reduction in 2030), 10.7 million ha of land needs to be transformed to SRP. Of these, 8.5 million ha are from agricultural land and 2.2 million ha are from grasslands (see Supporting Information Figure S4). In 2100, land requirements increase to 15.7 million ha, with 14.9 million ha from agricultural land and 0.8 million ha from grasslands (see Supporting Information Figure S5).

#### 3.3.2 Forestland Management Intensity

In forests, in scenarios that reduce biodiversity loss, there is globally more area left unmanaged, and in managed forests, biomass is produced with less intensity. Globally, there is more area left unmanaged in scenarios with the biodiversity constraint (see Figure S6). By shifting some of the biomass production to SRP, less biomass is produced in forests and therefore the model can increase areas left as secondary forests. This is called set-aside management or strict protection management. In general, for biodiversity, unmanaged forests are better than managed forests independent of the level of management intensity.

**Figure 4:**
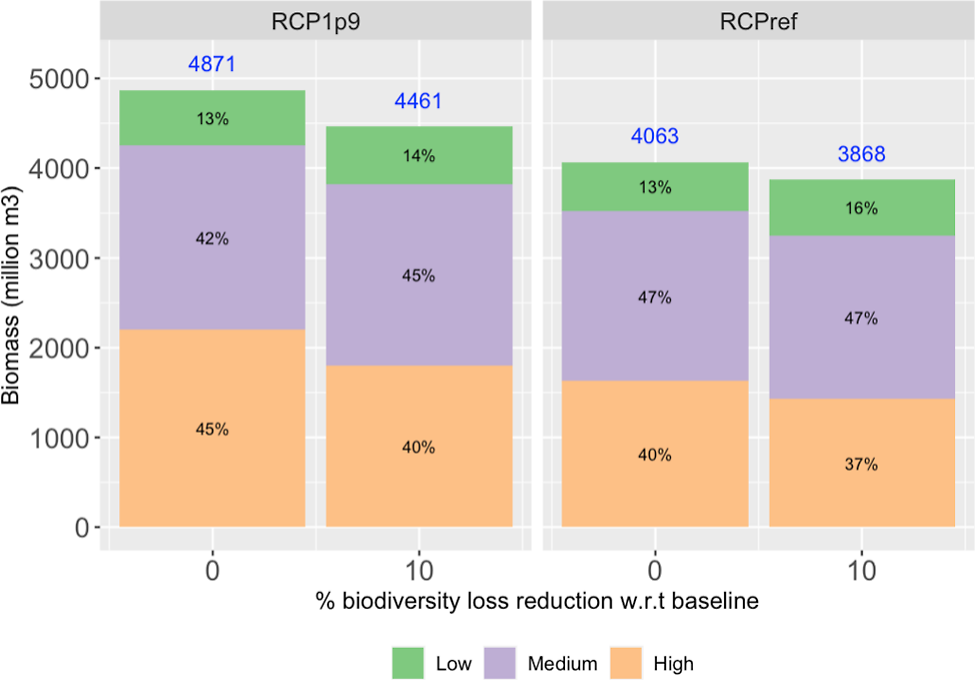
Contribution of each management intensity to biomass produced in forests for the scenarios analyzed, on a world basis, for 2030. Recall biomass produced in forests includes both biomass for bioenergy and biomass for wood products.

Globally, for the reduction of biodiversity loss, there is less biomass being obtained under high- and medium-intensity management. For biomass being produced in forests, the reduction in management intensity can be seen in two ways. First, in terms of areas, there is less area under medium and high intensity (see Figure S6). Second, in terms of harvest volumes under each intensity management (see Figure 4). Considering the reduced production of biomass in forests (numbers in blue in Figure 4) and the changes in the contribution of each intensity to the total production (the percentages in Figure 4), there is a reduction in the biomass volumes being produced under medium- and high-intensity management, and an increase in harvest volumes being produced using low-intensity management. This reduction in management intensity in forests is called close-to-nature management or low-level protection management.

#### 3.3.3 Location of Biomass Production

**Figure 5:**
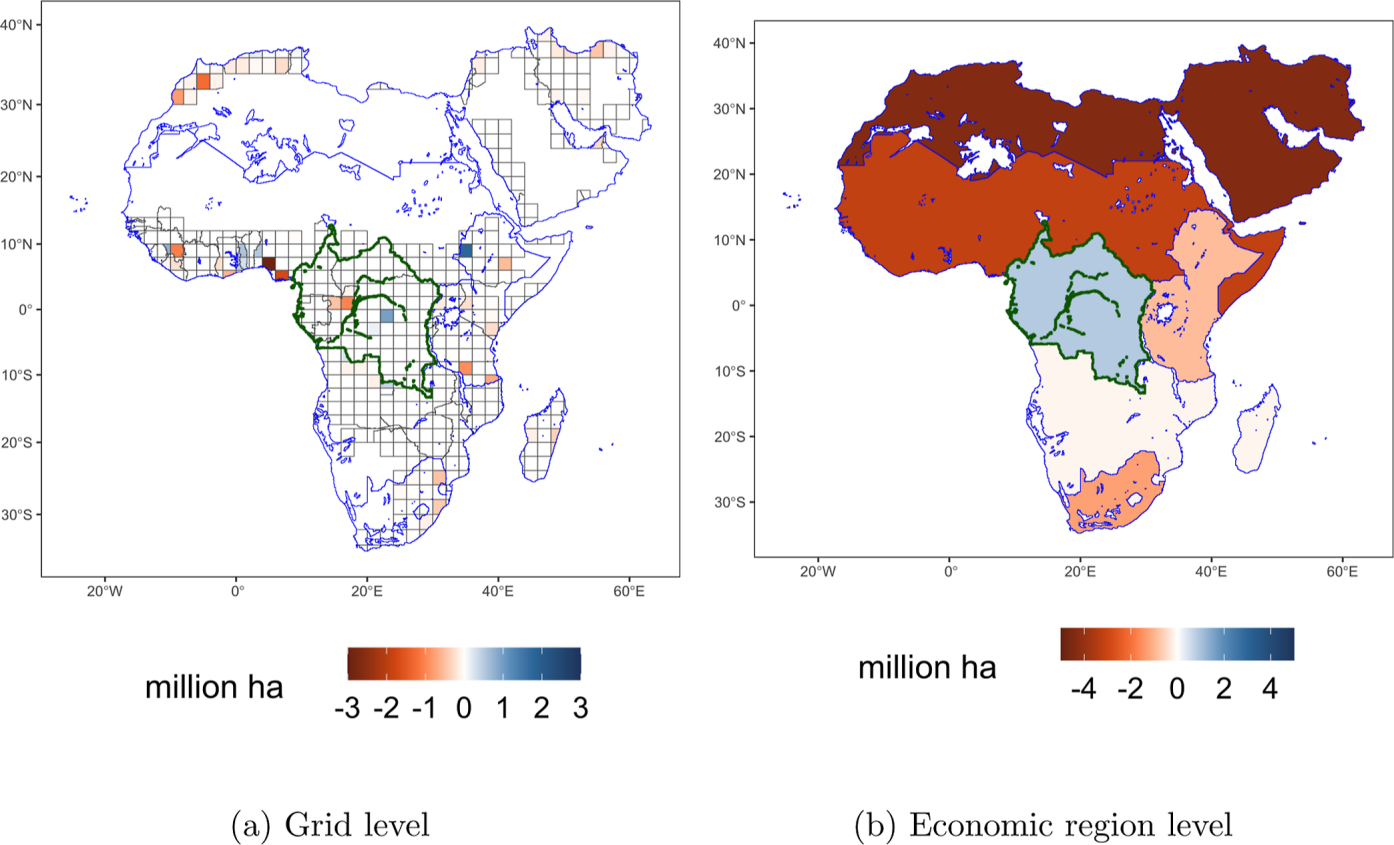
Example of reallocation of biomass production *within* and *between* economic regions. Differences in areas under high-intensity management for RW biomass production, with respect to the baseline without biodiversity constraint, and for economic regions in Africa and the Middle East, are presented. Blue indicates more areas under high-intensity management. Orange indicates less areas under high-intensity management. In green is the Congo Basin economic region highlighted. The reallocation of biomass production occurs *within* regions (shown in panel (a)) and *between* regions (shown in panel (b)). Panel (b) is the result of aggregating grid cell-level decisions for each economic region.

The model reallocates the production of biomass within and between economic regions. *Within* regions, the model reallocates production according to the 200 × 200 km grid. Figure 5 shows an example of how production areas under high-intensity management change inside the Congo Basin region (in green) for RCP1.9, under the 10% reduction in biodiversity impacts scenario in 2030. Figure 5a shows the grid cells on which the production decisions are being made. In blue, those grids where areas under high-intensity management will increase, and in orange those grids for which the same indicator will decrease. White grids indicate no change and the areas of the map without grids are those excluded from the analysis because forests cannot grow in these areas.

When aggregating the grid cell results for each economic region, the net effect on production areas is obtained. Figure 5b shows the example of the Congo Basin, which has a net reduction in areas under high-intensity management, which is consistent with the net reduction in RW biomass production as presented in Figure 2. With the aggregated results for each economic region, the *between regions* reallocation becomes evident. This can be seen in Figure 2 (via production volumes) and in Figure 5b (via areas under high-intensity management). For example, under the RCP1.9, 10% reduction scenario for 2030, the Former USSR, Congo Basin, and Canada increased production volumes (ordered by magnitudes). On the other hand, Brazil, USA, RCAM, India, Western Africa, and Argentina have reduced production.

By reallocating where biomass is produced the model can consider increased production in areas with less biodiversity impacts and reduce production in areas with higher biodiversity impacts. However, to identify the best locations, the model also considers yields and costs. Costs include biomass harvest cost, biomass transportation cost, biomass processing cost, and forest management change cost.

## 4 Sensitivity

The way biodiversity impacts are included in the mathematical formulation of the optimization model has underlying ethical assumptions^21^. To test how the main outcomes of the model would vary with different biodiversity implementations, we ran the model with three additional formulations of the biodiversity constraint. We end up with four different implementations: (1) *regional per taxa*—the original implementation (eq 4), (2) *global per taxa*—with a constraint per taxa, but using an estimation of global species loss (eq S15), (3) *regional all*—with regional species loss but with only one constraint that aggregates the biodiversity impacts of all taxa (eq S17), and (4) *regional per taxa-eco*—with a constraint per taxon and ecoregion using regional species loss (eq S18).

We find that on an aggregated world level, differences in biodiversity impacts do not vary much among the first three implementations. RW production reductions are similar between *regional per taxa* and *global per taxa*, and smaller for *regional all*. Shadow prices are higher for *global per taxa*, followed by *regional per taxa* and *regional all*. We find that *regional all* is the one with the highest biodiversity impacts, the least reduction in RW production, and the smallest shadow price. The lower shadow price also explains why this is the only implementation that was feasible for a 20% reduction scenario. These results for the *regional all* implementation come from the increased flexibility for the model to satisfy the constraint by interchanging impact both among ecoregions and taxa. The opposite in terms of flexibility is the *regional per taxa-eco* implementation, which was found infeasible under the current assumptions of the GLOBIOM-Forest model. This means that under these assumptions, it is not possible to reduce biodiversity loss for each pair ecoregion and taxon. Methodological details and results for this sensitivity analysis can be found in the Supporting Information Section S11.

## 5 Discussion

Because forests are important carbon sinks, there has been interest in future forest cover and carbon sequestration potential. A lot of the research has focused on studying how different policies, for example, the inclusion of a carbon tax, could change the amount and type of forests^10,28−32^.

Even with concerns about changes from natural to managed forests, less attention has been placed on studying policies to reduce biodiversity impacts when considering the potential of forests for carbon sequestration^9^. It is said that the magnitude of the biodiversity impacts in forests will not only depend on the future forest area, but on the type of forests and management used^7,8^. For future mitigation scenarios with increased demand for biomass, there have been ex-post analyses to investigate future impacts on biodiversity^5,6,33^ of different management practices. All these studies use ex-post analysis, in which biodiversity impacts are estimated, for future land covers and management practices from different scenarios obtained using decision-making models.

In contrast, in this study we go one step further and include biodiversity into the decision-making model, allowing us to identify better solutions, those with less biodiversity impacts and similar economic or climate mitigation outcomes. To do this, we integrated the economic model GLOBIOM-Forest, for forest management decisions, and the ecologic cSAR model, to represent biodiversity outcomes. We used the integrated model to analyze eight scenarios that intend to reduce biodiversity impacts by up to 40%, while guaranteeing the production of the woody biomass required for two climate change mitigation scenarios (RCPref and RCP1.9).

With this framework, we are able to identify trade-offs, challenges, and possibilities for improvement. We show that there is a potential trade-off between climate change mitigation and biodiversity conservation when woody biomass is used for large-scale bioenergy deployment scenarios. However, when incorporating biodiversity into decision making, it is possible to use forest management to mitigate the trade-off. We found that it is possible to reduce 10% of the biodiversity loss, associated with forests, with minor changes in the volumes of RW biomass being produced.

To achieve this reduction in biodiversity impacts, the model (1) shifts biomass production away from forests and into energy crops, (2) for biomass still produced in forests, it decreases the management intensity, and (3) it reallocates the biomass production between and within regions considering its biodiversity impacts, costs, and yields.

We show how global results, for both biodiversity impacts and biomass production, could differ from regional results. Similarly, we discussed differences in impacts between taxa and how biodiversity impacts could differ depending on how the biodiversity constraint is introduced.

It is important to mention the limitations of this study. The economic model GLOBIOM-Forest, as used here, has some key assumptions that affect the results. By not including deforestation and afforestation, the trade-off with climate change is incompletely assessed. RCP1.9 is characterized by high levels of bioenergy demand, but also by decreased deforestation. Thus, there could be important benefits of the high mitigation scenario on biodiversity that are not contemplated here. Also, since GLOBIOM-Forest has a simplified representation of the agricultural sector, it does not include the full opportunity cost of transforming agricultural lands or grasslands to energy crops. If included, this could affect the cost of shifting biomass production from forests to energy crops.

Besides limitations associated with the specific assumptions mentioned above, GLOBIOM-Forest, as similar models of its kind^b^ is subject to high levels of uncertainty. Sources of uncertainty include (1) parameter uncertainty, (2) model uncertainty, (3) algorithmic error, (4) coding error, and (5) scientific error^34^. For parameter uncertainty, Bertsimas and Den Hertog^35^ categorized it into measurement error, estimation error, implementation error, and errors due to inexact data. None of these sources of uncertainty are explicitly covered here due to computational limitations and the scope of the paper. However, this is an important aspect to be considered when interpreting the results.

Since the objective of this paper is to include biodiversity in the forest management decision-making model and assess how forest management decisions change, we could consider the impacts of these sources of uncertainty in two different components. The first component would be the GLOBIOM-Forest indicator results, such as the harvest volumes, the location and amounts of areas under each forest management, and trade amounts, among others. These results could be affected by these sources of uncertainty even if biodiversity is not included. The second component will be how the biodiversity impacts would change. This would include the magnitudes of the impacts, the trade-off between climate change mitigation and biodiversity, and the mechanisms that the model uses to alleviate this trade-off.

Regarding the first component and concentrating on parametric uncertainty, there are two main, complementary approaches used to deal with uncertainty: decision-making under uncertainty and Monte Carlo methods. Decision-making under uncertainty deals with the question: how to take optimal decisions considering the uncertainty of those parameters? Tools such as stochastic optimization, adaptive optimization, and robust optimization are used for this. For GLOBIOM, there has been work on a two-stage stochastic programming model^36−38^ that considers robust decision making in response to uncertain yields or water supply. To integrate biodiversity in this stochastic GLOBIOM model, it will be required to extend the level of detail of the existing versions and resolve current computational challenges, which was out of the scope of this paper.

The Monte Carlo methods instead focus on the questions: how does input uncertainty propagate to output uncertainty? What sources of uncertainty influence more the outcomes? And therefore which are more important uncertainties to reduce?^34^ In this regard, to the best of our knowledge, no such uncertainty analysis has yet been published for GLOBIOM or its GLOBIOM-Forest version.

Regarding the second component, that of how the conclusions derived from incorporating biodiversity may change, it is expected that biodiversity impact magnitudes would change in response to some input parameters. From the original GLOBIOM-Forest model, yields and bioenergy demands are expected to be the major components. With respect to biodiversity modeling, uncertainties in affinities are expected to have a major role as shown by ref (21). Of course, this would need to be validated with an explicit sensitivity analysis, to assess the magnitude of the changes. Even so, overall trends, such as the high mitigation scenario having higher impacts or the mechanisms used by the model to reduce biodiversity loss, should remain unchanged. This is important, as the interest of our study is not to predict future values, but to identify the possibilities for improvement, the trade-offs, the challenges, and overall to understand the relationships of the different components in the system.

Model resolution can be investigated. GLOBIOM-Forest can run with a 50 km × 50 km resolution, as well as at the level used here 200 km × 200 km, yet the capability of high-resolution modeling needs to be carefully gauged with respect to the resolution of data and the computational limitations.

Moving on to the biodiversity model (cSAR), there are several limitations as stated by ref (21) when detailing their proposed methodology to integrate cSAR in optimization models. Biodiversity is in itself a very complex concept that cannot be represented with only one indicator. Because of this, the integration of one indicator as done here is a starting point and a topic for methodological improvement. Besides, cSAR model limitations include that it omits that species can migrate once the habitat is lost, that the model does not indicate *which* species are being affected and *when*, that it fails to include the effects of habitat fragmentation, and since it only measures one dimension of biodiversity, that it does not account for population levels. More detail on these limitations can be seen in refs (21, 39 and 40). Moreover, there are limitations associated with the implementation of the cSAR model with GLOBIOM-Forest. Due to a lack of data, (1) the *Sorg* parameter included the number of species in all natural ecosystems and not only in forests, therefore overestimating the number of species committed to extinction; (2) secondary forests were assigned an affinity value of 1, indicating that species may be as good in secondary forests as they would be in their natural habitats. This may not be true, especially if the secondary forest is just beginning its natural recovery process. (3) It was assumed that the taxa in ecoregions in the same continent have the same affinity values. (4) There is important uncertainty about the affinities, especially for some combinations of taxon, management type, and ecoregion. For those combinations with missing information, an average for the same taxon, intensity, and continent was used. As discussed before and as shown by ref (21), this could have important implications in the magnitudes of the results.

Equally important is to note that we focused on the biodiversity impacts on forests due to habitat loss and omitted the potential benefits on biodiversity from climate change mitigation. Some studies have attempted to evaluate both the impacts of habitat loss and climate change, with contradictory results. Ohasi et al. and Di Marco et al. discuss on an ex-post basis how beneficial effects on biodiversity of mitigating climate change could be larger than negative effects due to land use change, for different SSPs and mitigation scenarios^41,42^. Yet, Hof et al., also using several mitigation scenarios, found that the negative impact of land-use change may surpass the benefit of climate change mitigation^4^. Hanssen et al. found that which effect is larger for biodiversity will depend on how early crop-based BECCS is deployed^43^. If considering 30 years, land use change may have a larger effect than climate change and if 80 years are considered, the reverse results are found. Therefore, considering climate change impacts on biodiversity could be a direction for future research that will further clarify the trade-off between climate change mitigation with high bioenergy deployment and biodiversity.

Future research should address some of these limitations. Dealing with parameter uncertainty is key for the robustness of the results and its use for policy making. This will include dealing with (1) the uncertainty in GLOBIOM-Forest without the incorporation of biodiversity and (2) with the uncertainty associated with the biodiversity modeling and its connection to the economic model. The latter would imply a sensitivity analysis of the biodiversity modeling parameters as the affinities and the slope of the cSAR curve. To do this, computational challenges must be overcome to reduce the running times of the model. Furthermore, including more drivers and dimensions of biodiversity can improve the representation of biodiversity impacts in decision making models. Finally, representing how biodiversity loss could affect forests’ ability to provide ecosystem services will incorporate biodiversity more fully into the IAM framework, showing how decision-making in the economic system could affect biodiversity, and also how biodiversity could affect our economic system^44^.

## Supporting Information Available

Additional methodological details (Sections S1, S5). Data Inputs (Sections S2, S3, S4, S6, S12). Additional results (Sections S7–S10). Sensitivity analysis details (Section S11) (PDF)

## Footnotes

a Currently, *Sorg*_*g*,*l*_ considers the reference number of species in all ecosystems in the ecoregion. For consistency, *Sorg*_*g*,*l*_ should be adjusted to only include the number of species that live in forests, suitable agricultural land, and suitable grasslands for SRP. However, this was not possible due to data limitations. This implies that the calculated number of species lost may be an overestimate.

b This includes land use change models, IAMs, CGEs, partial equilibrium models, and overall optimization models that are used to analyze future scenarios associated with climate change mitigation.

## References

[ref1] Hill S. L. L.; Gonzalez R.; Sanchez-Ortiz K.; Caton E.; Espinoza F.; Newbold T.; Tylianakis J.; Scharlemann J. P. W.; De Palma A.; Purvis A. Worldwide Impacts of Past and Projected Future Land-Use Change on Local Species Richness and the Biodiversity Intactness Index. bioRxiv 2018, 10.1101/311787.

[ref2] Chaudhary A.; Mooers A. Terrestrial Vertebrate Biodiversity Loss under Future Global Land Use Change Scenarios. Sustainability 2018, 10, 2764 10.3390/su10082764.

[ref3] Heck V.; Gerten D.; Lucht W.; Popp A. Biomass-Based Negative Emissions Difficult to Reconcile with Planetary Boundaries. Nat. Clim. Change 2018, 8, 151–155. 10.1038/s41558-017-0064-y.

[ref4] Hof C.; Voskamp A.; Biber M. F.; Bohning-Gaese K.; Engelhardt E. K.; Niamir A.; Willis S. G.; Hickler T. Bioenergy Cropland Expansion May Offset Positive Effects of Climate Change Mitigation for Global Vertebrate Diversity. Proc. Natl. Acad. Sci. U.S.A. 2018, 115, 13294–13299. 10.1073/pnas.1807745115. 30530689 PMC6310845

[ref5] Schulze K.; Malek Z.; Verburg P. H. The Impact of Accounting for Future Wood Production in Global Vertebrate Biodiversity Assessments. Environ. Manage. 2020, 66, 460–475. 10.1007/s00267-020-01322-4. 32627082 PMC7434756

[ref6] Giuntoli J.; Barredo J. I.; Avitabile V.; Camia A.; Cazzaniga N. E.; Grassi G.; Jasinevičius G.; Jonsson R.; Marelli L.; Robert N.; Agostini A.; Mubareka S. The Quest for Sustainable Forest Bioenergy: Win-Win Solutions for Climate and Biodiversity. Renewable Sustainable Energy Rev. 2022, 159, 112180 10.1016/j.rser.2022.112180.

[ref7] Chaudhary A.; Burivalova Z.; Koh L. P.; Hellweg S. Impact of Forest Management on Species Richness: Global Meta-Analysis and Economic Trade-Offs. Sci. Rep. 2016, 6, 23954 10.1038/srep23954. 27040604 PMC4819217

[ref8] Griscom B. W.; Goodman R. C.; Burivalova Z.; Putz F. E. Carbon and Biodiversity Impacts of Intensive Versus Extensive Tropical Forestry. Conserv. Lett. 2018, 11, e12362 10.1111/conl.12362.

[ref9] Ferreira J.; Lennox G. D.; Gardner T. A.; Thomson J. R.; Berenguer E.; Lees A. C.; Mac Nally R.; Aragão L. E. O. C.; Ferraz S. F. B.; Louzada J.; et al. Carbon-Focused Conservation May Fail to Protect the Most Biodiverse Tropical Forests. Nat. Clim. Change 2018, 8, 744–749. 10.1038/s41558-018-0225-7.

[ref10] Austin K. G.; Baker J. S.; Sohngen B. L.; Wade C. M.; Daigneault A.; Ohrel S. B.; Ragnauth S.; Bean A. The Economic Costs of Planting, Preserving, and Managing the World’s Forests to Mitigate Climate Change. Nat. Commun. 2020, 11, 5946 10.1038/s41467-020-19578-z. 33262324 PMC7708837

[ref11] Sohngen B.; Mendelsohn R. An Optimal Control Model of Forest Carbon Sequestration. Am. J. Agric. Econ. 2003, 85, 448–457. 10.1111/1467-8276.00133.

[ref12] Sohngen B.; Mendelsohn R.; Sedjo R. Forest Management, Conservation, and Global Timber Markets. Am. J. Agric. Econ. 1999, 81, 1–13. 10.2307/1244446.

[ref13] Lauri P.; Forsell N.; Di Fulvio F.; Snäll T.; Havlik P. Material Substitution Between Coniferous, Non-Coniferous and Recycled Biomass—Impacts on Forest Industry Raw Material Use and Regional Competitiveness. For. Pol. Econ. 2021, 132, 102588 10.1016/j.forpol.2021.102588.

[ref14] Lauri P. GLOBIOM-Forest Documentation. 2021. https://github.com/iiasa/GLOBIOM_foreswebt (accessed June 27, 2022).

[ref15] Pereira H. M.; Daily G. C. Modeling Biodiversity Dynamics in Countryside Landscapes. Ecology 2006, 87, 1877–1885. 10.1890/0012-9658(2006)87[1877:MBDICL]2.0.CO;2. 16937624

[ref16] Pereira H. M.; Ziv G.; Miranda M. Countryside Species–Area Relationship as a Valid Alternative to the Matrix-Calibrated Species–Area Model. Conserv. Biol. 2014, 28, 874–876. 10.1111/cobi.12289. 24673576 PMC4262074

[ref17] Olson D. M.; Dinerstein E.; Wikramanayake E. D.; Burgess N. D.; Powell G. V. N.; Underwood E. C.; D’amico J. A.; Itoua I.; Strand H. E.; Morrison J. C.; et al. Terrestrial Ecoregions of the World: A New map of Life on earth. BioScience 2001, 51, 933–938. 10.1641/0006-3568(2001)051[0933:teotwa]2.0.co;2.

[ref18] Proença V.; Pereira H. M. Species–Area Models to Assess Biodiversity Change in Multi-Habitat Landscapes: The Importance of Species Habitat Affinity. Basic Appl. Ecol. 2013, 14, 102–114. 10.1016/j.baae.2012.10.010.

[ref19] Chaudhary A.; Verones F.; de Baan L.; Hellweg S. Quantifying Land Use Impacts on Biodiversity: Combining Species-Area Models and Vulnerability Indicators. Environ. Sci. Technol. 2015, 49, 9987–9995. 10.1021/acs.est.5b02507. 26197362

[ref20] Chaudhary A.; Brooks T. M. Land Use Intensity-Specific Global Characterization Factors to Assess Product Biodiversity Footprints. Environ. Sci. Technol. 2018, 52, 5094–5104. preprint version: http://dx.doi.org/10.2139/ssrn.4803736 10.1021/acs.est.7b05570. 29648805

[ref21] Azuero-Pedraza C. G.; Thomas V. Incorporating Biodiversity Impacts in Land Use Decisions. Ecol. Model. 2023. In Review.

[ref22] Curran M.; Maia de Souza D.; Anton A.; Teixeira R. F.; Michelsen O.; Vidal-Legaz B.; Sala S.; Mila i Canals L. How Well Does LCA Model Land Use Impacts on Biodiversity?—A Comparison with Approaches from Ecology and Conservation. Environ. Sci. Technol. 2016, 50, 2782–2795. 10.1021/acs.est.5b04681. 26830787

[ref23] de Baan L.; Mutel C. L.; Curran M.; Hellweg S.; Koellner T. Land Use in Life Cycle Assessment: Global Characterization Factors Based on Regional and Global Potential Species Extinction. Environ. Sci. Technol. 2013, 47, 9281–9290. 10.1021/es400592q. 23875861

[ref24] Fricko O.; Havlik P.; Rogelj J.; Klimont Z.; Gusti M.; Johnson N.; Kolp P.; Strubegger M.; Valin H.; Amann M.; et al. The Marker Quantification of the Shared Socioeconomic Pathway 2: A Middle-Of-The-Road Scenario for the 21st Century. Global Environ. Change 2017, 42, 251–267. 10.1016/j.gloenvcha.2016.06.004.

[ref25] Rogelj J.; Popp A.; Calvin K. V.; Luderer G.; Emmerling J.; Gernaat D.; Fujimori S.; Strefler J.; Hasegawa T.; Marangoni G.; et al. Scenarios Towards Limiting Global Mean Temperature Increase Below 1.5 °C. Nat. Clim. Change 2018, 8, 325–332. 10.1038/s41558-018-0091-3.

[ref26] Riahi K.; van Vuuren D. P.; Kriegler E.; Edmonds J.; O’Neill B. C.; Fujimori S.; Bauer N.; Calvin K.; Dellink R.; Fricko O.; et al. The Shared Socioeconomic Pathways and Their Energy, Land Use, and Greenhouse Gas Emissions Implications: An Overview. Global Environ. Change 2017, 42, 153–168. 10.1016/j.gloenvcha.2016.05.009.

[ref27] van Vuuren D. P.; Riahi K.; Moss R.; Edmonds J.; Thomson A.; Nakicenovic N.; Kram T.; Berkhout F.; Swart R.; Janetos A.; Rose S. K.; Arnell N. A Proposal for a New Scenario Framework to Support Research and Assessment in Different Climate Research Communities. Global Environ. Change 2012, 22, 21–35. 10.1016/j.gloenvcha.2011.08.002.

[ref28] Favero A.; Mendelsohn R. The Land-Use Consequences of Woody Biomass with More Stringent Climate Mitigation Scenarios. J. Environ. Prot. 2017, 08, 61–73. 10.4236/jep.2017.81006.

[ref29] Favero A.; Daigneault A.; Sohngen B. Forests: Carbon Sequestration, Biomass Energy, or Both?. Sci. Adv. 2020, 6, eaay6792 10.1126/sciadv.aay6792. 32232153 PMC7096156

[ref30] Thomson A. M.; Calvin K. V.; Chini L. P.; Hurtt G.; Edmonds J. A.; Bond-Lamberty B.; Frolking S.; Wise M. A.; Janetos A. C. Climate Mitigation and the Future of Tropical Landscapes. Proc. Natl. Acad. Sci. U.S.A. 2010, 107, 19633–19638. 10.1073/pnas.0910467107. 20921413 PMC2993329

[ref31] Kindermann G. E.; Obersteiner M.; Rametsteiner E.; McCallum I. Predicting the Deforestation-Trend Under Different Carbon-Prices. Carbon Bal. Manag. 2006, 1, 15 10.1186/1750-0680-1-15. PMC1766350 17150095

[ref32] Daigneault A.; Baker J. S.; Guo J.; Lauri P.; Favero A.; Forsell N.; Johnston C.; Ohrel S. B.; Sohngen B. How the Future of the Global Forest Sink Depends on Timber Demand, Forest Management, and Carbon Policies. Global Environ. Change 2022, 76, 102582 10.1016/j.gloenvcha.2022.102582. PMC10631560 38024226

[ref33] Rosa F.; Di Fulvio F.; Lauri P.; Felton A.; Forsell N.; Pfister S.; Hellweg S. Can Forest Management Practices Counteract Species Loss Arising from Increasing European Demand for Forest Biomass under Climate Mitigation Scenarios?. Environ. Sci. Technol. 2023, 57, 2149–2161. 10.1021/acs.est.2c07867. 36706339 PMC9910049

[ref34] Gillingham K.; Nordhaus W.; Anthoff D.; Blanford G.; Bosetti V.; Christensen P.; McJeon H.; Reilly J. Modeling Uncertainty in Integrated Assessment of Climate Change: A Multimodel Comparison. J. Assoc. Environ. Resour. Econ. 2018, 5, 791–826. 10.1086/698910.

[ref35] Bertsimas D.; Den Hertog D. Robust and Adaptive Optimization; Dynamic Ideas LLC: Belmont, MA, 2022.

[ref36] Ermolieva T. Y.; Ermoliev Y. M.; Havlik P.; Mosnier A.; Leclere D.; Kraksner F.; Khabarov N.; Obersteiner M. Systems Analysis of Robust Strategic Decisions to Plan Secure Food, Energy, and Water Provision Based on the Stochastic Globiom Model. Cybern. Syst. Anal. 2015, 51, 125–133. 10.1007/s10559-015-9704-2.

[ref37] Ermolieva T.; Havlík P.; Ermoliev Y.; Mosnier A.; Obersteiner M.; Leclère D.; Khabarov N.; Valin H.; Reuter W. Integrated Management of Land Use Systems under Systemic Risks and Security Targets: A Stochastic Global Biosphere Management Model. J. Agric. Econ. 2016, 67, 584–601. 10.1111/1477-9552.12173.

[ref38] Ermolieva T.; Havlik P.; Ermoliev Y.; Khabarov N.; Obersteiner M. Robust Management of Systemic Risks and Food-Water-Energy-Environmental Security: Two-Stage Strategic-Adaptive GLOBIOM Model. Sustainability 2021, 13, 857 10.3390/su13020857.

[ref39] Verones F.; Hellweg S.; Antón A.; Azevedo L. B.; Chaudhary A.; Cosme N.; Cucurachi S.; de Baan L.; Dong Y.; Fantke P.; et al. LC-IMPACT: A Regionalized Life Cycle Damage Assessment Method. J. Ind. Ecol. 2020, 24, 1201–1219. 10.1111/jiec.13018.

[ref40] de Baan L.; Alkemade R.; Koellner T. Land Use Impacts on Biodiversity in LCA: A Global Approach. Int. J. Life Cycle Assess. 2013, 18, 1216–1230. 10.1007/s11367-012-0412-0.

[ref41] Ohashi H.; Hasegawa T.; Hirata A.; Fujimori S.; Takahashi K.; Tsuyama I.; Nakao K.; Kominami Y.; Tanaka N.; Hijioka Y.; Matsui T. Biodiversity can Benefit from Climate Stabilization Despite Adverse Side Effects of Land-Based Mitigation. Nat. Commun. 2019, 10, 5240 10.1038/s41467-019-13241-y. 31748549 PMC6868141

[ref42] Di Marco M.; Harwood T. D.; Hoskins A. J.; Ware C.; Hill S. L. L.; Ferrier S. Projecting Impacts of Global Climate and Land-Use Scenarios on Plant Biodiversity Using Compositional-Turnover Modelling. Global Change Biol. 2019, 25, 2763–2778. 10.1111/gcb.14663. 31009149

[ref43] Hanssen S. V.; Steinmann Z. J. N.; Daioglou V.; Čengić M.; Van Vuuren D. P.; Huijbregts M. A. J. Global Implications of Crop-Based Bioenergy with Carbon Capture and Storage for Terrestrial Vertebrate Biodiversity. GCB Bioenergy 2021, 14, 307–321. 10.1111/gcbb.12911. 35875590 PMC9299942

[ref44] Tschirhart J. Integrated Ecological-Economic Models. Annu. Rev. Resour. Econ. 2009, 1, 381–407. 10.1146/annurev.resource.050708.144113.

[ref45] Havlík P.; Schneider U. A.; Schmid E.; Böttcher H.; Fritz S.; Skalský R.; Aoki K.; Cara S. D.; Kindermann G.; Kraxner F.; Leduc S.; McCallum I.; Mosnier A.; Sauer T.; Obersteiner M. Global Land-Use Implications of First and Second Generation Biofuel Targets. Energy Pol. 2011, 39, 5690–5702. 10.1016/j.enpol.2010.03.030.

[ref46] Havlik P.; Valin H.; Herrero M.; Obersteiner M.; Schmid E.; Rufino M. C.; Mosnier A.; Thornton P. K.; Bottcher H.; Conant R. T.; Frank S.; Fritz S.; Fuss S.; Kraxner F.; Notenbaert A. Climate Change Mitigation Through Livestock System Transitions. Proc. Natl. Acad. Sci. U.S.A. 2014, 111, 3709–3714. 10.1073/pnas.1308044111. 24567375 PMC3956143

