# Supplementary material for: Managing Forests for Biodiversity Conservation and Climate Change Mitigation
Source: Environ Sci Technol. 2024 May 14;58(21):9175–86. doi: 10.1021/acs.est.3c07163 (PMC11137864; doi:10.1021/acs.est.3c07163)
Supplement: Supplementary file 1 — es3c07163_si_001.pdf [file es3c07163_si_001.pdf]

# Supporting Information: Managing forests for biodiversity conservation and climate change mitigation

Cindy G. Azuero-Pedraza[1,2,3,4] Pekka Lauri[2] Andrey Lessa Derci Augustynczik [2] Valerie M. Thomas [1,5]

[1] H. Milton Stewart School of Industrial and Systems Engineering, Georgia Institute of Technology, Atlanta, GA 30332, USA

[2] International Institute for Applied Systems Analysis (IIASA), Schlossplatz 1, A-2361 Laxenburg, Austria

[3] CMCC Foundation - Euro-Mediterranean Center on Climate Change, Via Marco Biagi 5, Lecce,73100, Italy

[4] RFF-CMCC European Institute on Economics and the Environment, Via Bergognone 34, Milan, 20144, Italy

[5] School of Public Policy, Georgia Institute of Technology, Atlanta, GA 30332, USA

## Summary

35 pages

14 figures

4 tables

# S1 Details on GLOBIOM-Forest model

GLOBIOM[1,2] is a land use change model that represents the interactions between the agricultural (including livestock), forestry, bioenergy, and fisheries & aquaculture sectors to understand and estimate land allocations and future land cover under different scenarios. It is a partial equilibrium model that follows a bottom-up approach, where supply is represented on a spatially explicit basis

(using the simulation units (SimU) that correspond to the intersection of countries, grid cells, altitude, slope and soil class), the demand is represented on a regional basis and bilateral trade between regions is included. Since it maximizes total surplus,[3] land allocation decisions are based on the profitability of the activities by land use type. It incorporates information from the EPIC agricultural model and is coupled with the G4M forest management model. The model is run recursively with 10 year time steps, from 2000 to 2100. GLOBIOM is used as the land module of two integrated assessment models, WITCH (from the RFF-CMCC European Institute on Economics and the Environment) and MESSAGEix (from the International Institute of Applied System Analysis (IIASA)).

GLOBIOM-Forest is a version of GLOBIOM that focuses on a more detailed representation of the forest sector, while simplifying the representation of the agricultural and bioenergy sectors. It is a bottom-up partial equilibrium model in which total economic surplus (see equation S14) is maximized. In it, spatially explicit supply-related decisions are made on spatial units that correspond to the intersection of a grid, that can be $200km \times 200km$ or $50km \times 50km$ resolution, with country boundaries. On the other hand, demand is represented on a regional basis (up to 58 regions - Table S4[4]). The model includes: (a) transportation costs of woody biomass from forest to mill gate within each region, (b) harvest costs, (c) process costs, (d) investment costs, (e) trade costs, and (f) land use change costs. a,b,f are spatially explicit and c,d,e are on a regional basis. The model includes a representation of both forestry and the forest industry. *Biomass production* is via the primary harvested products (pulplogs, sawlogs, industrial plantations biomass, other industrial roundwood, fuelwood, logging residues) and one non-harvested product (deadwood). *Forest industry* is represented via the by-products (sawdust, woodchips, bark, black liquor, recycled wood, recycled paper, recycled pulp), the intermediate products (chemical pulp, mechanical pulp), and final products (sawnwood, plywood, fiberboard, other industrial roundwood, newsprint, paper for printing or writing, packaging, other paper, fuelwood, energy wood).

Regarding the production of biomass, GLOBIOM-Forest decides on (1) the *area* of forest to be harvested during the rotation period for each spatial unit and forest management type and (2)

[3] Which is a monetary measure of welfare.

[4] For the results presented here, the model was run with 58 regions that include 180 countries.

the harvested *quantities* of a particular primary product in each spatial unit and under each forest management type. In comparison to GLOBIOM, GLOBIOM-Forest includes more than one forest management intensity (Low, Medium and High), has detail on tree species (distinguishing between coniferous (softwood) and non-coniferous (hardwood)) and includes details on age-class dynamics. Management intensities are defined as a combination of assumptions on (1) the percentage of the increment that is harvested, (2) the limit on logging residues that can be obtained and (3) a minimum amount of the increment that has to be left as deadwood.

Regarding the production of intermediate and final demand products, the model decides on (1) the quantity of final products to produce by processing primary products and (2) the processing capacity of the main final products. It also decides on the level of investment in each region and each product, which will increase the production capacity.

GLOBIOM-Forest also includes bilateral trade of forest products between regions, deciding the quantities of each product that are exported or imported from region to region.

Energy crops are incorporated through short rotation plantations (SRP). These are represented separately from the previously mentioned forest management types because by a sustainability assumption energy crops are not located in forestland[5]. Instead, the model decides the amount of area devoted to these industrial plantations by transforming from natural land[6], grasslands or cropland. The representation of these land use changes is simplified in this version compared to GLOBIOM. The biodiversity impact of both forest management and land use transformations to SRPs are represented in this paper.

Biomass for energy can be produced both in forestland and in SRPs. However, the rest of the biomass for forest products can only be produced in forestland.

GLOBIOM-Forest is a recursive optimization model that is calibrated from years 2000 to 2020, and runs in ten-year intervals up to 2100. Data sources for calibration include the Global Forest Resource Assessment (FRA) which provides regional data on forest types and harvest potential between coniferous and non-coniferous tree species; the World Database on Protected Areas (WDPA)

[5]It has been observed that when forests are converted to SRP, it usually decreases the biomass stock per ha, whereas when cropland or managed grassland are converted to SRP, biomass stock per ha usually increases.

[6]For the results presented here, the model was not allowed to transform natural land to SRP. Natural land includes other natural habitats different from forests and other lands not actively being managed.

for grid level data on forest management and Nature Map Explorer. These three databases were used to improve the allocation of forest and forest management areas during the calibration period. Additionally, the FAOSTAT database was used for reference volumes for demand functions, forest industry production capacities, the separation between coniferous and non-coniferous final products, harvest volumes and net trade quantities. Finally, BACI trade data was used for the bilateral trade quantities. Other sources of data, besides those for calibration purposes, include G4M which provides increments, harvest costs, and total forest area as a result of deforestation and afforestation decisions.

The model is run under the Shared Socioeconomic Pathway 2 (SSP2) - the middle of the road scenario for global socioeconomic development to 2100[3,4]. Within this pathway, two RCPs are explored: RCP1.9, representing a scenario in which global warming is kept below 1.5°C by keeping the increase in radiative forcing to less than 1.9 W/m2 in 2100[5], and RCP7.0, the reference scenario for MESSAGE in which increased radiative forcing reaches 7 W/m2 by 2100, here called RCPref. In the model, the SSP affects the GDP and population data which then affect the demand functions. The RCPs affect the bioenergy and wood pellet demand as in[6].

# S2 Data Mappings

Three data components have to be considered, the spatial units, the forest management types and the time periods.

**Spatial units** As described in biodiversity model description section 2.2, the biodiversity impact indicator is calculated on an ecoregion basis. On the other hand, the data for affinities in the biodiversity model (from[7]), is on a continental level (excluding Antarctica). This requires a mapping between continents and ecoregions. The assignation of an ecoregion to a continent is through countries, therefore an initial mapping between ecoregions and countries is required. This was done in ArcGIS using the UIA World Country Boundaries Layer and the WWF ecoregions layer. It is assumed that the affinity value $h$ for the ecoregion will correspond to the affinity value assigned to the continent to which the ecoregion belongs. If an ecoregion has area in more than

one country, a weighted average of the affinity factors of the continents was calculated, based on the proportion of the ecoregion area in each continent.

The GLOBIOM-Forest model was run on a 200km x 200km ($2^{o}$) grid resolution for all countries. The spatial units in GLOBIOM-Forest correspond to the intersection between country boundaries and this grid. To connect the ecoregion level and these spatial units, an intersection of the two layers was done in ArcGIS. From this, a mapping was created. It includes (1) if the combination between the ecoregion and the spatial unit exists, and (2) the weight $mW_{s,j}$ used in equations 3, S1, S2 and S3.

**Management Types**[7] contains information on the response ratios, and therefore affinities, for ten management types, whereas GLOBIOM-Forest includes three management intensity types. The mapping used is presented in Table S1. See Table S3 for the definition of each forest management type in GLOBIOM-Forest.

Table S1: Mapping between[7] and GLOBIOM-Forest management types

| [7] management type | GLOBIOMf management type |
|---|---|
| Clear-cutting | High |
| Retention | Low |
| Selection system | Low |
| Selective logging | Medium |
| Reduced Impact Logging (RIL) | Low |
| Plantation-timber | High |
| Plantation-fuel | High |
| Plantation-non timber | Not apply |
| Agroforestry | Not apply |
| Slash & Burn | Not apply |

It is assumed that affinity factors will not change between tree species (coniferous and non-coniferous) and the affinity factor for a GLOBIOM-Forest management intensity will correspond to the average of the[7] management types according to mapping in Table S1.

**Land use Types**[8] contains information to estimate the affinities for seven land use types. The following table show the mapping used to connect to GLOBIOM-Forest land use types for SRP.

Table S2: Mapping between[8] and GLOBIOM-Forest land use types

| [8] land use type | GLOBIOMf land use type |
|---|---|
| Annual-crops | Agricultural land |
| Pastures | Grassland |
| Permanent crops | SRP |
| Natural habitat* | Other natural land |

* Natural habitat, by definition, have affinities values of 1.

**Time periods** The biodiversity cSAR model estimates potential regional species loss by comparing a reference scenario to a future scenario. To combine this with the 10-year periods (from 2000-2100) of GLOBIOM-Forest, the "pristine" reference scenario remains the same for all GLOBIOM-Forest time periods, while the future scenario corresponds to each of the GLOBIOM-Forest runs. This means that the potential species loss estimated is always with respect to the reference scenario.

# S3 Mapping for management intensities

Table S3: Description of forest management types in GLOBIOM forest when deciding on forest land types

| Name | ID GLOBIOMf | Decision Type | Description |
|---|---|---|---|
| Primary forest | PriFor | Forest Management | Unmanaged forest. Correspond to FAO primary forests, which are forests without human impact. |
| Secondary forest | Cur0 | Forest Management | Unmanaged forest. Correspond to the forest area that is not a primary forest nor a production forest. Its a forest with human impact, but not used for production. |
| Low intensity management | CurC_L, CurNC_L | Forest Management | Managed forest with 50% of maximum increment harvested for EU and 25% of maximum increment harvest for the rest of the world (ROW). Its a mix of several low intensity management types: Retention forestry, "Nature" management, Selective logging with uneven age management.<br>Requirement of deadwood from remaining increment= 0<br>Logging residues share= 0 |
| Medium intensity management | CurC_M, CurNC_M | Forest Management | Managed forest with 75% of maximum increment harvested for EU and 50% of maximum increment harvested for rest of the world (ROW). Could be considered Multifunctional management.<br>Requirement of deadwood from remaining increment= 0<br>Logging residues share= 0.25 |
| High intensity management | CurC, CurNC | Forest management | Managed forest with 100% of maximum increment is harvested. Its a mix of several high intensity management types: Planted forest, clear-cut management, even aged monoculture.<br>Requirement of deadwood from remaining increment= 0<br>Logging residues share = 0.5 |

In tableS3, the maximum increment available for harvest comes from a G4M estimate of the mean annual increment (MAI) based on Net Primary Production (NPP) maps and the normal forest assumption[9].

# S4 Bioenergy demands for the two climate change mitigation scenarios

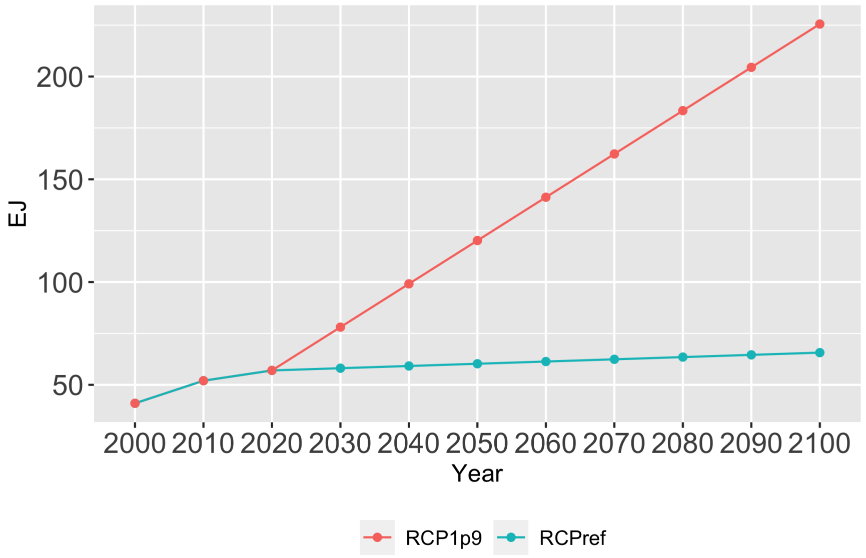


Figure S1: Global bioenergy demand for each climate mitigation scenario.

# S5 Mathematical formulation changes to GLOBIOM-Forest

The required changes to incorporate biodiversity to the partial equilibrium model mathematical formulation via the constraint approach[10] are explained next. It is important to note that the constraint on biodiversity was activated after the GLOBIOM-Forest calibration period, i.e., for 2030-2100.

Sets:

Defining the following sets according to GLOBIOM-Forest,

$S$: set of spatial units in GLOBIOM-Forest indexed in $s$ *.

$F$: set of forest management types in GLOBIOM-Forest indexed in $i$. Includes {Primary forest, Secondary forest, Low intensity management, Medium intensity management, High intensity management}

$U$: set of land use types with area suitable for SRP from GLOBIOM-Forest indexed in $i'$. Includes {Agricultural land, Grassland, Other natural land}

Add the following sets:

$G$: set of taxonomic groups indexed in $g$. Includes {Mammals, Birds, Amphibians, Plants}.

$L$: set of ecoregions of the world indexed in $l$.

* In GLOBIOM-Forest, the spatial units correspond to the intersection of the indices that correspond to the sets COUNTRY, ALLCOLROW, AltiClass, SLPCLASS, SOILCLASS and AEZCLASS. ALLCOLROW represents the gridcell which can be defined and used under two different resolutions, 200km x 200km (2°) or 50km x 50km (0.5°). The other sets represent Altitude, Slope, Soil and Agro-ecological zones (AEZ), respectively.

<u>Parameters:</u>

Add the following parameters:

$Sorg_{g,l}$: Number of species of taxa $g$ present in ecoregion $l$ in the reference scenario.

$Aorg_l$: Natural habitat area in the reference scenario in ecoregion $l$. See assumptions in the biodiversity model description section 2.2.

$z_l$: The slope of the log-log plot of the power law that describes how rapidly species are lost as habitat is lost in SAR models. Defined for each ecoregion $l$.

$h\_FM_{g,i,l}$: SAR model parameter that reflects the affinity of taxonomic group $g$ to management type $i$ in ecoregion $l$.

$h\_LU_{g,i',l}$: SAR model parameter that reflects the affinity of taxonomic group $g$ to land use type $i'$ in ecoregion $l$.

$h\_SRP_{g,l}$: SAR model parameter that reflects the affinity of taxonomic group $g$ to SRP in ecoregion $l$.

$Bmax_g$: Maximum number of species allowed to disappear regionally across the ecoregions, due to habitat loss caused by forest management decisions, for each taxon $g$.

$mW_{s,l}$: Weight of spatial unit $s$ in ecoregion $l$. Based on area.

Decision variables from GLOBIOM-Forest:

The decision variables from GLOBIOM-Forest that will be connected with the biodiversity model Countryside SAR are:

$L_{s,i}$ =HARVEST_VAR(COUNTRY, ALLCOLROW, AltiClass, SLPCLASS, SOILCLASS, AEZCLASS, ForMngType)

This variable ($L_{s,i}$) represents the area of forest that will be harvested in each spatial unit under each forest management type during the rotation time, measured in 1000 ha.

$SRP_{s,i'}$ =SRP_VAR(COUNTRY,ALLCOLROW,AltiClass,SLPCLASS, SoilClass, AEZCLASS, LC_TYPE_SRP)

This variable ($SRP_{s,i'}$) represents the area changed from suitable land use type $i'$ to SRP in spatial unit $s$, measured in 1000 ha.

Auxiliary variables:

$A\_FM_{i,l}$: Area under each forest management type $i$ in each ecoregion $l$ in the scenario being analyzed.

$A\_LU_{i',l}$: Suitable area for SRP under each land use type $i'$ in each ecoregion $l$ in the scenario being analyzed.

$A\_SRP_l$: Area under SRP in each ecoregion $l$ in the scenario being analyzed.

$Slost^{Regional}_{g,l}$: corresponds to the calculation from the countryside SAR model for taxon $g$ and ecoregion $l$. It represents the potential **regional** species loss due to habitat loss.

Constraints:

1. Defines $A\_FM_{i,l}$. Adds over the spatial units that belong to each ecoregion.

$$A\_FM_{i,l} = \sum_s L_{s,i} \cdot mW_{s,l},\ \forall i \in F, l \in L \tag{S1}$$

2. Defines $A\_LU_{i',l}$. Adds over the spatial units that belong to each ecoregion.

$$A\_LU_{i',l} = \sum_s (SRP\_DATA_{s,i'} - SRP_{s,i'}) \cdot mW_{s,l},\ \forall i' \in U, l \in L \tag{S2}$$

3. Defines $A\_SRP_l$. Adds over the spatial units that belong to each ecoregion.

$$A\_SRP_l = \sum_s SRP_{s,i'} \cdot mW_{s,l},\ \forall l \in L \tag{S3}$$

4. Defines $Slost_{g,l}$ auxiliary variable.

$$Slost_{g,l}^{Regional} = Sorg_{g,l}\left[1 - \left(\frac{\sum_{i\in F} h\_FM_{g,i,l}A\_FM_{i,l} + \sum_{i'\in U} h\_LU_{g,i',l}A\_LU_{i',l} + h\_SRP_{g,l}A\_SRP_l}{Aorg_l}\right)^{z_l}\right], \quad \forall g \in G, l \in L \tag{S4}$$

5. *Constraint methodology* Defines an upper limit for biodiversity loss of current forest management allocation for each taxon

$$\sum_{l\in L} Slost_{g,l}^{Regional} \leq Bmax_g \, \forall g \in G \tag{S5}$$

**About $Bmax_g$** For the results presented here $Bmax_g$ is a function of the total biodiversity loss per taxa, calculated on an ex-post basis, for the baseline scenario (without the incorporation of biodiversity). First, the baseline model is run. Second, using the results for the harvest areas under each type of management ($HARVEST\_VAR$) and the amount of area transformed to SRP ($SRP\_VAR$), the biodiversity impact for baseline model ($Bmax_{0_g}$) is estimated using the same cSAR model representation that is then used in the model that includes biodiversity. Third, $Bmax_g$ is defined according to equation S6.

$$Bmax_g = (1 - \%)Bmax_{0_g} \tag{S6}$$

where % corresponds to the percentage of reduction desired. Here 10%, 20%, 30% and 40% were tested. For the regional-per taxa implementation only 10% was mathematically feasible. The baseline scenario, without the biodiversity constraint, is represented in this document by a 0% desired reduction.

**Piecewise linear approximation for cSAR** For computational efficiency, GLOBIOM-Forest is modeled as an LP. With constraint S4 the model becomes non-linear. The following are the changes used to linearize the problem by using a piecewise linear approximation for the countryside SAR model.

Add the following set, parameter, auxiliary variables, and constraints:

Set:

$K$: $\{1, 2, ..., m\}$ Number of breakpoints for linearization.

Parameter:

$a_k$: Breakpoints for linearization of $y = cSAR1_{g,l}^{z_l}$ where $a_1 < a_2 < ... < a_m$. $a_1 = 0$ and $a_m = 1$.

Auxiliary variables:

$cSAR1_{g,l}$: Ratio between areas available for species relationship on SAR model.

$t_{k,g,l}$: Weight given to break point $k$ for each combination of ecoregion $l$ and taxon $g$.

Constraints:

1. Defines $cSAR1$. $cSAR1$ is defined over interval $[0, 1]$.

$$cSAR1_{g,l} = \frac{\sum_{i \in F} h\_ForMng_{g,i,l} A\_ForMng_{i,l} + \sum_{i' \in U} h\_LU_{g,i',l} A\_LU_{i',l} + h\_SRP_{g,l} A\_SRP_l}{Aorg_l}$$

$$\forall g \in G, l \in L \tag{S7}$$

Note that with constraint S7, the countryside SAR model (constraint S4) can be rewritten as,

$$Slost_{g,l} = Sorg_{g,l} \left[1 - (cSAR1_{g,l})^{z_l}\right] \; \forall g \in G, l \in L \tag{S8}$$

2. Implement linearization. Replacing $cSAR1_{g,l}^{z_l}$ in equation S8 with a piecewise linear approximation $L(cSAR1_{g,l}^{z_l})$.

$$Slost_{g,l} = Sorg_{g,l}[1 - L(cSAR1_{g,l}^{z_l})] \; \forall g \in G, l \in L \tag{S9}$$

where the linearization $L(cSAR1_{g,l}^{z_l})$ is defined as:

$$L(cSAR1_{g,l}^{z_l}) = \sum_{k=1}^{m} a_{k,g,l}^{z_l} t_{k,g,l} \tag{S10}$$

3. Define $cSAR1_{g,l}$ as the convex combination of break points.

$$cSAR1_{g,l} = \sum_{k=1}^{m} a_{k,g,l} t_{k,g,l}, \qquad \forall g \in G, l \in L \tag{S11}$$

$$\sum_{k=1}^{m} t_{k,g,l} = 1, \qquad \forall g \in G, l \in L \tag{S12}$$

4. Define the lower bound on $t_{k,g,l}$

$$t_{k,g,l} \geq 0, \ \forall g \in G, l \in L, k = \{1, 2, ..., m\} \tag{S13}$$

As mentioned in[10], since $y = cSAR1^{z_l}$ is a concave function when $0 \leq h \leq 1$, then it is guaranteed that no more than two consecutive weights can be nonzero. The decision on $m$ implies a trade-off between model accuracy and the computational time to run. As expected, with increased breakpoints, the linear approximation becomes a better representation of the non-linear cSAR function, but because of the increased number of $t_{k,g,l}$ decision variables, computational time increases significantly when $m$ increases. We defined $m = 6$ and the domain of cSAR1 as $[a_1 = 0, a_6 = 1]$.

# S6 GLOBIOM-Forest regions

In GLOBIOM-Forest, the model includes 180 countries that could be grouped into 58 regions to facilitate the analysis of the results. Table S4.

Table S4: Regions and associated countries in GLOBIOM forest

| Region Identifier | Region Name | Countries |
|---|---|---|
| AUTReg | Austria | Austria |
| BELReg | Belgium | Belgium |
| BGRReg | Bulgaria | Bulgaria |

Table S4: continued

| Region Identifier | Region Name | Countries |
|---|---|---|
| CYPReg | Cyprus | Cyprus |
| CZEReg | Czech Republic | Czech Republic |
| DEUReg | Germany | Germany |
| DNKReg | Denmark | Denmark |
| ESPReg | Spain | Spain |
| ESTReg | Estonia | Estonia |
| FINReg | Finland | Finland |
| FRAReg | France | France |
| GBRReg | UK | UK |
| GRCReg | Greece | Greece |
| HUNReg | Hungary | Hungary |
| IRLReg | Ireland | Ireland |
| ITAReg | Italy | Italy |
| LTUReg | Lithuania | Lithuania |
| LUXReg | Luxembourg | Luxembourg |
| LVAReg | Latvia | Latvia |
| MLTReg | Malta | Malta |
| NLDReg | Netherlands | Netherlands |
| POLReg | Poland | Poland |
| PRTReg | Portugal | Portugal |
| ROUReg | Romania | Romania |
| SVKReg | Slovakia | Slovakia |
| SVNReg | Slovenia | Slovenia |
| SWEReg | Sweden | Sweden |
| HRVReg | Croatia | Croatia |
| ANZ | Australia New Zealand | Australia, New Zealand |
| BrazilReg | Brazil | Brazil |
| CanadaReg | Canada | Canada |
| ChinaReg | China | China |
| CongoBasin | Congo Basin | Cameroon, Central Africa Republic, Congo Democratic Republic, Republic of the Congo, Equatorial Guinea, Gabon |
| Former_USSR | Former USSR | Armenia, Azerbaijan, Belarus, Georgia, Kazakhstan, Kyrgyzstan, Moldova Republic, Russian Federation, Tajikistan, Turkmenistan, Uzbekistan |
| IndiaReg | India | India |
| JapanReg | Japan | Japan |
| MexicoReg | Mexico | Mexico |
| MidEastNorthAfr | Middle East North Africa | Algeria, Bahrain, Egypt, Iran, Iraq, Israel, Jordan, Kuwait, Lebanon, Libya, Morocco, Oman, Palestin, Qatar, Saudi Arabia, Syria, Tunisia, United Arab Emirates, West Sahara, Yemen |

Table S4: continued

| Region Identifier | Region Name | Countries |
|---|---|---|
| Pacific_Islands | Pacific Islands | Fiji Islands, French Polynesia, New Caledonia, Papua New Guinea, Samoa, Solomon Islands, Vanuatu |
| RCAM | Rest of Central America | Bahamas, Belize, Costa Rica, Cuba, Dominican Republic, El Salvador, Guadeloupe, Guatemala, Haiti, Honduras, Jamaica, Nicaragua, Panama, Trinidad and Tobago |
| RCEU | Rest of Central Eastern Europe | Albania, Bosnia and Herzegovina, Macedonia, Serbia-Montenegro |
| ROWE | Rest of Western Europe | Greenland, Iceland, Switzerland |
| NorwayReg | Norway | Norway |
| RSAM | Rest of South America | Bolivia, Chile, Colombia, Ecuador, Falkland Islands, French Guiana, Guyana, Paraguay, Peru, Suriname, Uruguay, Venezuela |
| RSAS | Rest of South Asia | Afghanistan, Bangladesh, Bhutan, Nepal, Pakistan, Sri Lanka |
| RSEA_OPA | Other Pacific Islands Rest | Brunei Darussalam, Myanmar, Philippines, Singapore, Thailand, Timor Leste |
| RSEA_PAC | Planned Asia Rest | Laos, Mongolia, Vietnam |
| SouthAfrReg | South Africa | South Africa |
| SouthKorea | South Korea | South Korea |
| EasternAf | Eastern Africa | Burundi, Ethiopia, Kenya, Rwanda, Tanzania, Uganda |
| SouthernAf | Southern Africa | Angola, Botswana, Comoros, Lesotho, Madagascar, Malawi, Mauritius, Mozambique, Namibia, Reunion, Swaziland, Zambia, Zimbabwe |
| WesternAf | Western Africa | Benin, Burkina Faso, Cape Verde, Chad, Cote d'Ivoire, Djibouti, Eritrea, Gambia, Ghana, Guinea, Guinea Bissau, Liberia, Mali, Mauritania, Niger, Nigeria, Senegal, Sierra Leone, Somalia, Sudan, Togo |
| TurkeyReg | Turkey | Turkey |
| USAReg | USA | USA |
| ArgentinaReg | Argentina | Argentina |
| IndonesiaReg | Indonesia | Indonesia |
| MalaysiaReg | Malaysia | Malaysia |
| UkraineReg | Ukraine | Ukraine |

# S7 Why don't economic outcomes change much?

There are small changes in economic outcomes, measured by biomass production in forests, for two reasons. First, to adapt to the imposed biodiversity constraint, the model uses three main strategies: (1) shifting biomass production to SRP, (2) reducing management intensity in managed forests, and (3) reallocating biomass production between and within regions. These strategies allow the model to supply almost the same levels of biomass while reducing the biodiversity impacts.

Second, demanded quantities do not change much. This is because endogenous demand for forest products for material use is highly inelastic $[-0.3, -0.1]$, and exogenous demand for bioenergy biomass must be satisfied. For endogeneous demands, on a global basis, the largest reduction in demanded quantities is of -4.53% for fuelwood biomass (FW_biomass_C) for the RCPref, 10% reduction scenario in 2030.

Similar results are obtained for the total global economic surplus, for more details see supplementary S8.1.

# S8 Total global surplus

The objective function of GLOBIOM-Forest is to maximize the total global economic surplus $CSPS$ according to

$$
\begin{aligned}
CSPS = & \underbrace{\sum_{a,k}\int_0^{x_{a,k}} D_{a,k}(x_{a,k})dx_{a,k}}_{\text{Total benefits}} - \underbrace{\sum_{a,i,j}\int_0^{z^f_{a,i,j}} C^{fmc}_{a,i,j}(z^f_{a,i,j})dz^f_{a,i,j}}_{\text{Total forest mngt costs}} - \underbrace{\sum_{a,b,k}\int_0^{e_{a,b,k}} C^{trade}_{a,b,k}(e_{a,b,k})de_{a,b,k}}_{\text{Total trading costs}} \\
& - \underbrace{\sum_{s,i} c^{harv}_{s,i} L_{s,i}}_{\text{Total harvest costs in forests}} - \underbrace{\sum_{s,i'} c^{harvSRP}_{s,i'} SRP_{s,i'}}_{\text{Total harvest costs in SRP}} - \underbrace{\sum_{a,i'}\int_0^{z_{a,i'}} C^{luc}_{a,i'}(z_{a,i'})dz_{a,i'}}_{\text{Total LUC costs}} \\
& - \underbrace{\sum_{s,i,k} c^{trans}_{s,i} y^f_{s,i,k}}_{\text{Total transportation costs}} - \underbrace{\sum_{a,k}\int_0^{y_{a,k}} C^{supply}_{a,k}(y_{a,k})dy_{a,k}}_{\text{Total supply costs}} - \underbrace{\sum_{a,f} c^{proc}_{a,f} y^p_{a,f}}_{\text{Total processing costs}} - \underbrace{\sum_{a,k} c^{inv}_{a,k} I_{a,k}}_{\text{Total investment costs}}
\end{aligned}
\tag{S14}
$$

where indices $a, b$ correspond to economic regions (See S6), $k$ are products, $s$ are spatial units, $i, j$ are forest management types, $f$ are forest industry production activities, and $i'$ are the land use types than can be transformed to SRP. The following are the decision variables: $x_{a,k}$ are demanded quantities, $y_{a,k}$ are supplied quantities per region, $y^p_{a,f}$ are the levels of production activities execution, $y^f_{s,i,k}$ are the supplied quantities per spatial unit under each management type, $e_{a,b,k}$ are traded quantities, $L_{s,i}$ are harvested areas ($HARVEST\_VAR$) in forests, $SRP_{s,i'}$ are the harvested areas in SRP (SRP_VAR), $z^f_{a,i,j}$ is the forest management area changed from $i$ to $j$, $z_{a,i'}$ is the land use change area from $i'$ to SRP, and $I_{a,k}$ are the investment quantities.

## S8.1 Why does the total global economic surplus have small changes?

On a global basis, total benefits and total costs do not change significantly. Total benefits depend on endogenous demands and endogenous demands are highly inelastic, as mentioned in a previous section S7, resulting in unchanged demanded quantities.

Total costs, as shown in equation S14 depend on several sub costs. Figure S2 shows how global cost components, change when the biodiversity constraint is introduced in 2030 for the scenario with 10% reduction for both climate change mitigation scenarios.

The graph shows how some costs increase while others decrease, resulting in a net small change in total costs. To exemplify this, note that SRP costs, forest management costs and trading costs increase, whereas harvest costs and transportation costs decrease. This is a result of producing less biomass in forests, more biomass in SRP, reallocating production, and changing forest management toward less intensification.

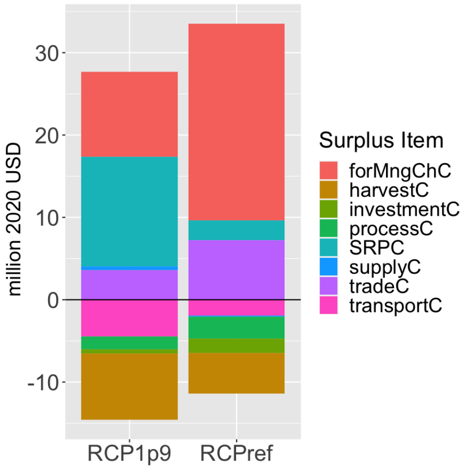


Figure S2: How cost components change for the mitigation scenarios and the introduction of the biodiversity loss constraint (for a 10% reduction) for 2030. SRP costs (SRPC) include both land use change cost and harvest cost in SRP.

# S9 Additional results for 2030 and 2100

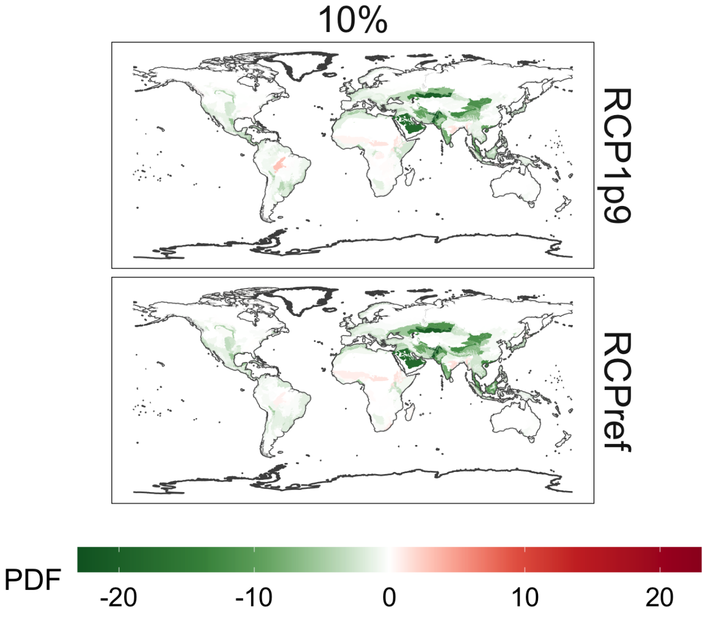


Figure S3: Potential regional species loss for mammals in 2030 under the biodiversity loss reduction scenario assessed on a regional basis.

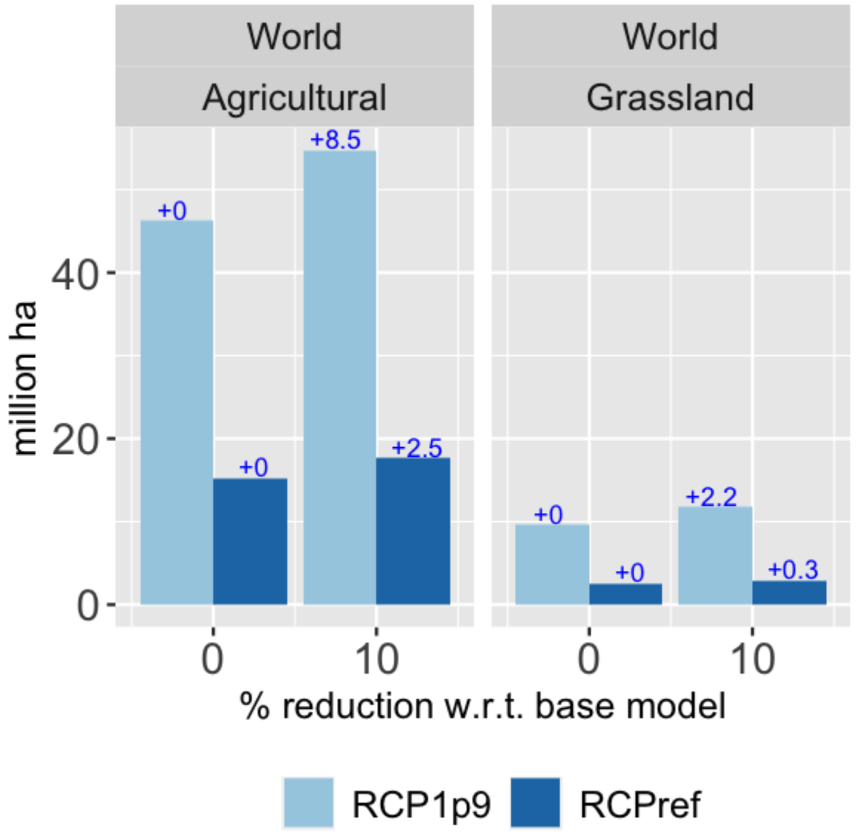


Figure S4: Land use change resulting from increased SRP, with its origin between agricultural land or grasslands, for the scenarios assessed, on a global basis, for 2030.

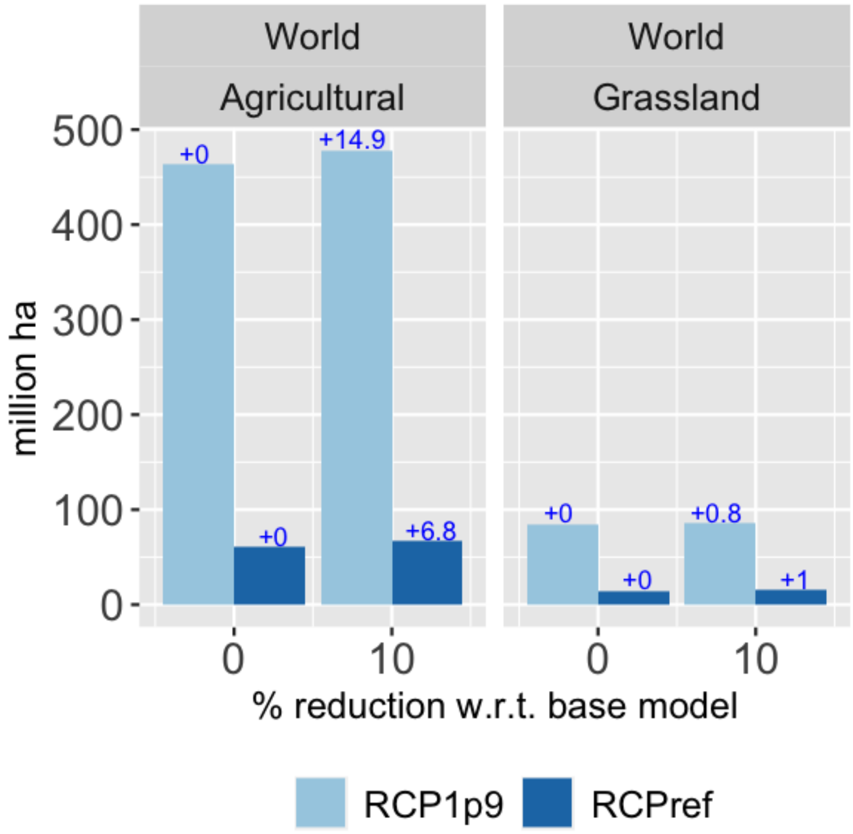


Figure S5: Land use change resulting from increased SRP, with its origin between agricultural land or grasslands, for the scenarios assessed, on a global basis, for 2100.

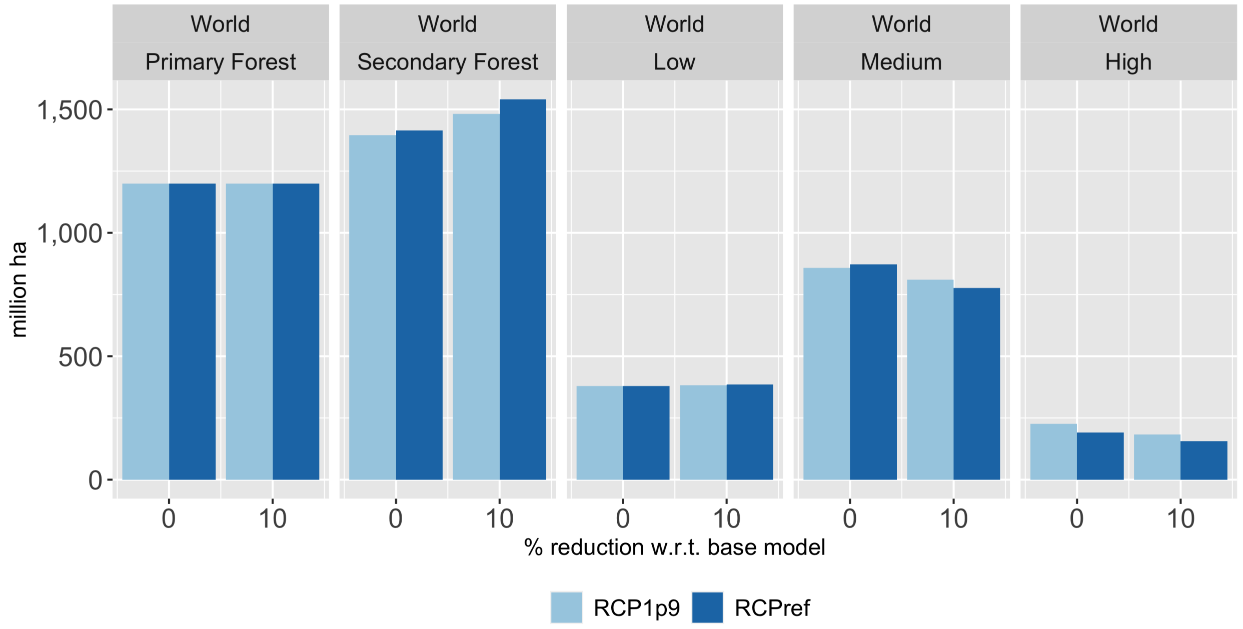


Figure S6: Forest areas under each management type, on a global basis, for the scenarios assessed in 2030. Unmanaged forests are Primary forests and Secondary forests. Managed forests are shown under low, medium and high intensity management.

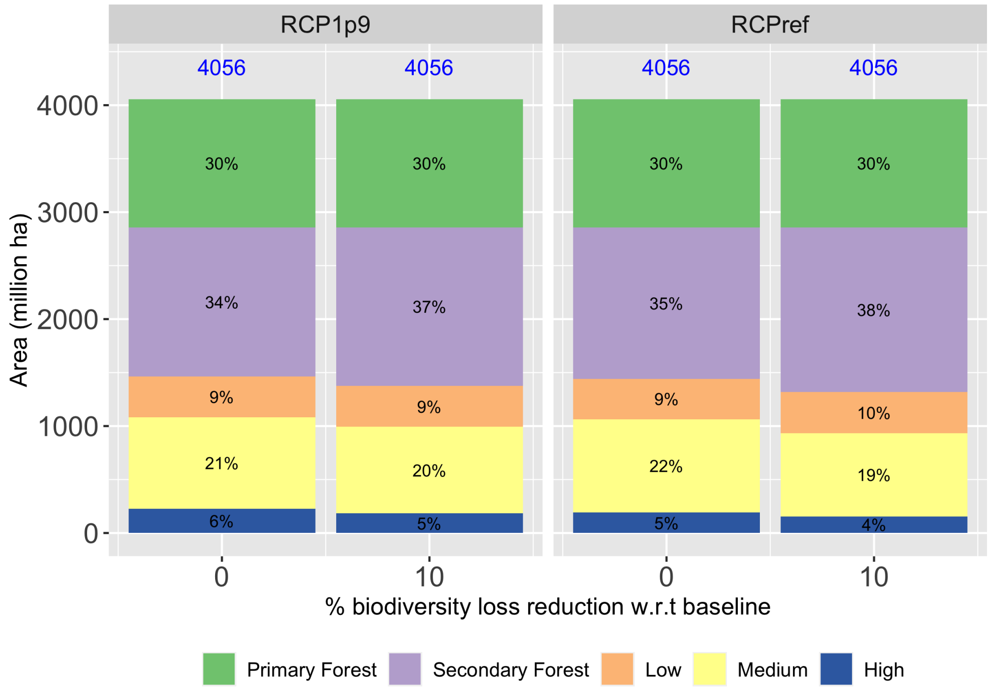


(a) 2030

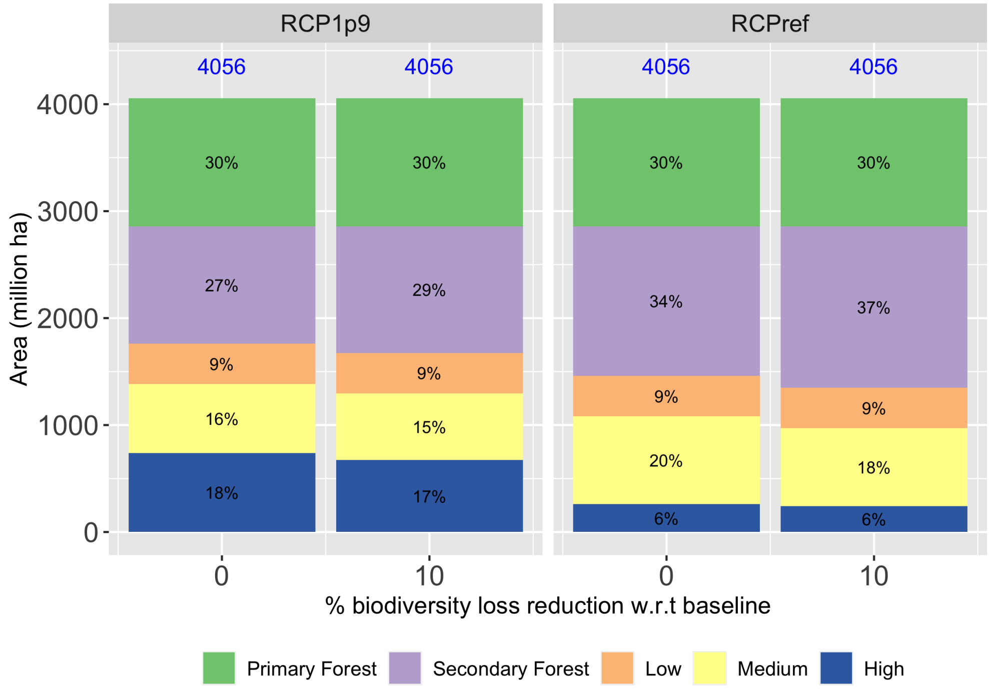


(b) 2100

Figure S7: Forest area under each management type, for 2030 and 2100, under the scenarios analyzed. Primary forest and secondary forest correspond to unmanaged forests and Low, Medium and High correspond to the three levels of intensity management in managed forests.

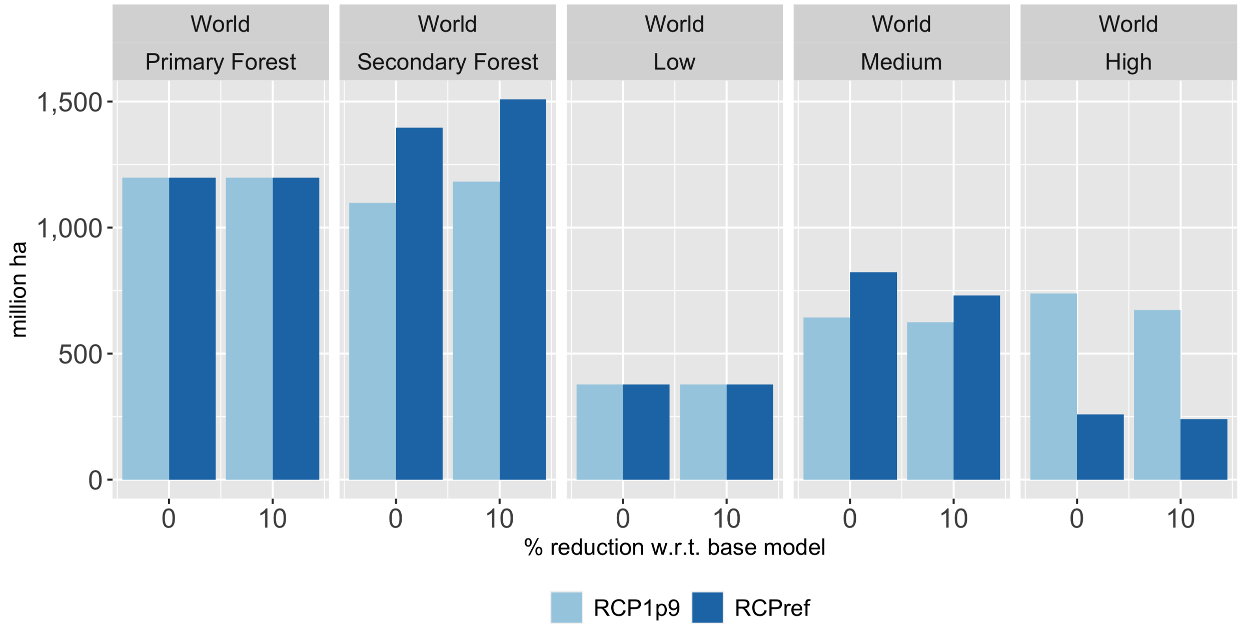


Figure S8: Forest areas under each management type, on a world basis, for the scenarios assessed in 2100. Unmanaged forests are Primary forests and Secondary forests. Managed forests are the ones under low, medium and high intensity

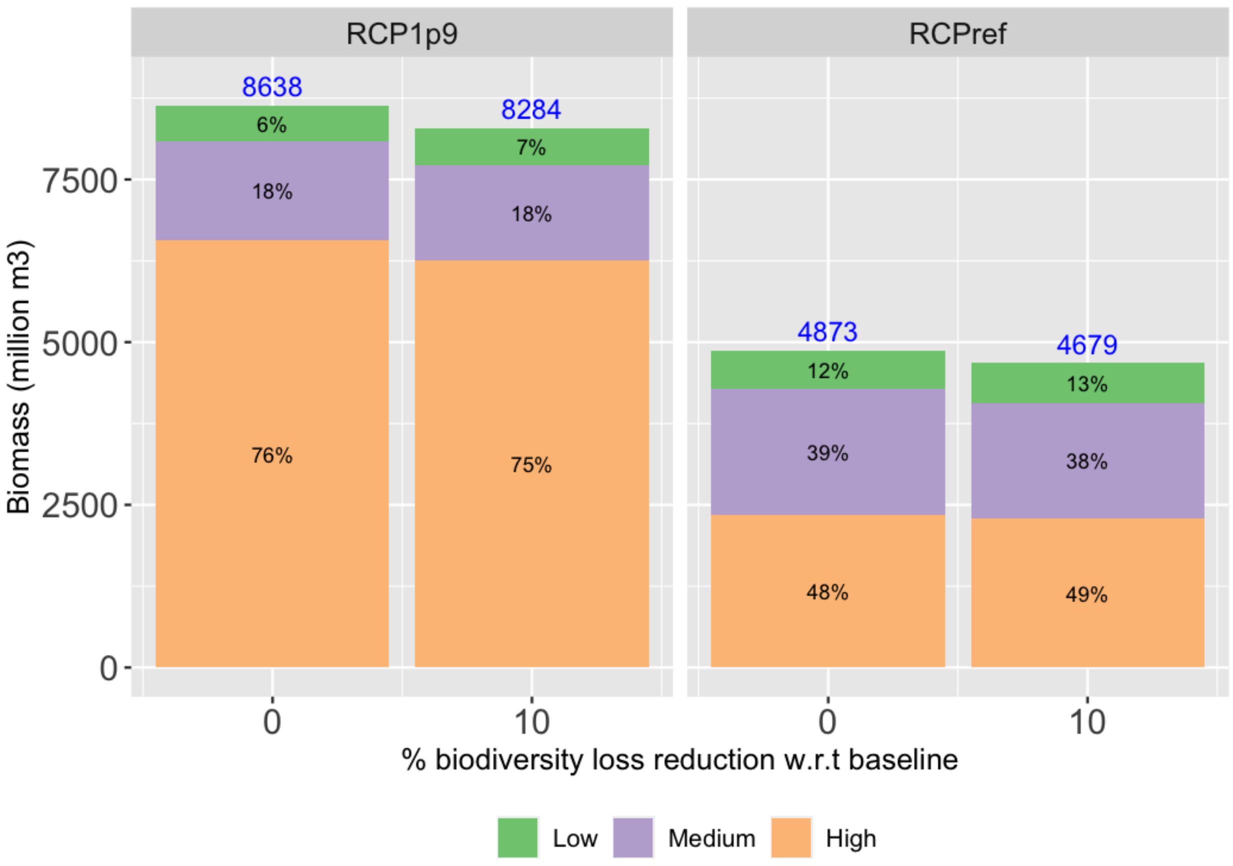


Figure S9: Contribution of each management intensity to biomass produced in forests for the scenarios analyzed, on a world basis, for 2100.

## S10 Carbon storage in forests outcomes

**Age class dynamics and carbon storage** GLOBIOM-forest includes spatial explicit age-class dynamics which determines available biomass for harvests and biomass stock of forests for each period. Each grid cell of the model includes different management systems with management specific growth curves and initial age-class distribution. The S-shaped growth curves are based on a Chapman-Richards biomass growth model similar to[11] and[12]. Initial age-class areas are based on[13]. At the grid level, growth curves and initial age-class distributions are calibrated to match biomass data from the G4M model[9,14].In addition to this, the model is calibrated to match FRA's[15] country level data on forest areas and biomass stocks. After 2020, the age-class dynamics develops endogenously based on periodic harvest volumes, growth curves, and mortality.

To maintain the sustainability of harvest potential over time in the recursive dynamics, the model includes two additional constraints. First, for each grid cell, it is not allowed to harvest more than what biomass grows. Second, it is not allowed to harvest age-classes that are younger than the optimal rotation time. Optimal rotation times vary in the range of 20-80 years depending on the management system and climate zone. For details on different forest management systems and model calibration see GLOBIOM-Forest documentation (https://github.com/iiasa/GLOBIOM_forest).

With this representation of age-classes, the carbon stored aboveground in forest biomass can be estimated. This is by using the forest area under each age-class and the biomass carbon stock per hectare (ha), which differs between planted and natural forests, and among the spatial location of the forests.

**Changes in carbon stored in forest when introducing biodiversity** On a global basis, carbon stored in aboveground biomass has small changes with the introduction of the biodiversity constraint. With respect with the baseline without including biodiversity, the maximum increase across scenarios is of 0.88% (2.5 PgC) for the RCP1.9, 40% reduction scenario in 2070 and the maximum reduction is of 0.27% (0.85 PgC) for the RCPref, 40% reduction scenario in 2040. See Figure S10.

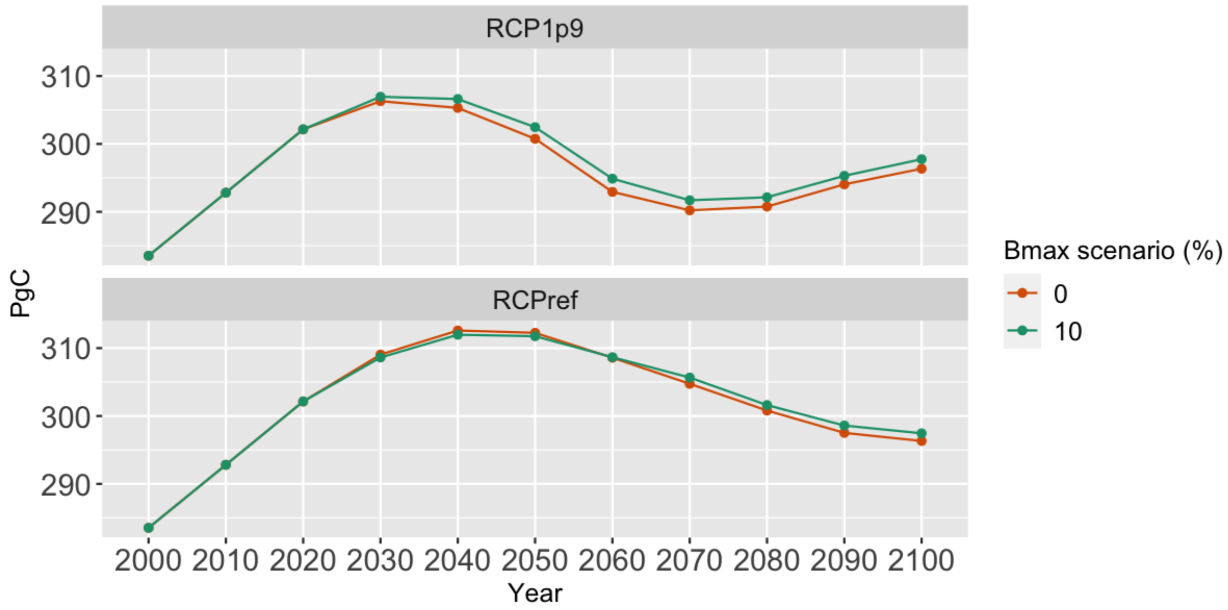


Figure S10: Carbon stored in aboveground biomass under the biodiversity scenarios considered from 2000 to 2100.

# S11 Sensitivity to biodiversity constraint implementation

Biodiversity impacts are sensitive to the way the biodiversity constraint is included. As mentioned by Azuero-Pedraza and Thomas[10], the way in which the biodiversity constraint is introduced has ethical implications. It also affects the incentives of the model to prioritize protecting primary and secondary forests between one ecoregion and the others. Following[10], we ran the model with three additional representations of the biodiversity constraint, resulting in a total of 4 different implementations. First, what we will call *Regional per taxa*, which corresponds to the original implementation (eq. 4) in this paper. Second, what we will call *Global per taxa* which corresponds to a constraint per taxa, but with global species loss (instead of regional), as shown in equation S15. $Slost_{g,l}^{Global}$ is calculated as presented in equation S16, which is based on[8] . In this equation, $VS_l$ is the vulnerability score of ecoregion $l$ and represents the level of endemism of the ecoregion. Vulnerability scores from Table S2 in[16]'s supplementary information are used.

$$\sum_{l \in L} Slost_{g,l}^{Global} \leq Bmax_g \, \forall g \in G \tag{S15}$$

$$Slost_{g,l}^{Global} = VS_l \cdot Slost_{g,l}^{Regional} \tag{S16}$$

Third, called *Regional all*, with regional species loss, but adding over all taxa instead of per taxa (as in equation S17).

$$\sum_{\substack{g \in G, \\ l \in L}} Slost_{g,l}^{Regional} \leq Bmax \tag{S17}$$

Fourth, called *Regional per taxa-eco*, in which we constraint biodiversity loss for each taxon and ecoregion (as in equation S18).

$$Slost_{g,l}^{Regional} \leq Bmax_{g,l} \, \forall g \in G, l \in L \tag{S18}$$

$Bmax$, $Bmax_g$ and $Bmax_{g,l}$ represent the limits on biodiversity loss on an aggregated level, per taxon, and per taxon & ecoregion accordingly. In Figure S11 we show the difference in biodiversity impacts between the first three ways to introduce the constraint for the RCP1.9 and 10% reduction scenario for 2030. *Regional per taxa-eco* is not presented in the figure because it was found that keeping all other assumptions in the model the same, this implementation, i.e. the reduction of biodiversity loss for each ecoregion and taxon, is infeasible.

The impact in the figure is presented as the total regional species loss or total sum of extirpations as a % of the number of species in the reference scenario $Sorg_{g,l}$ by taxa, i.e. $\sum_{l \in L} Slost_{g,l}^{Regional} / \sum_{l \in L} Sorg_{g,l} \forall g \in G$.

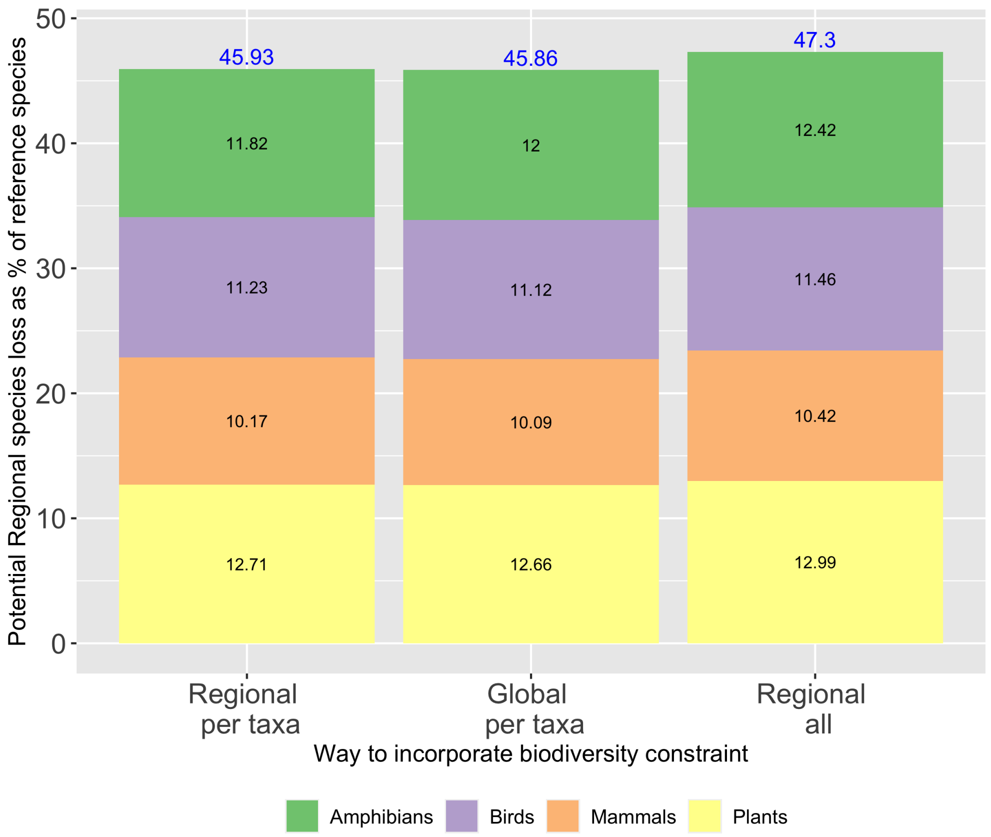


Figure S11: Comparison between three ways to restrict biodiversity loss, for the RCP1.9 and 10% reduction scenario for 2030. Regional per taxa is used in this study. The biodiversity impact is presented as total potential regional species loss (total sum of extirpations) as a percentage of the reference number of species included in the model for each taxon.

In this figure, when comparing between *Global per taxa* and *Regional per taxa*, we can see the effect of including the level of endemism of the ecoregion in the constraint. Because of how the level of endemism is included in equation S16, i.e. by multiplying $VS_l$, it was expected that the model would not have the same incentives to protect biodiversity in regions with lower levels of endemism (those with $VS_l$ very close to zero) in comparison to using the *Regional per taxa* implementation. For those ecoregions with very low levels of endemism, for the model, the regional biodiversity impact becomes irrelevant to satisfy the constraint under the *Global per taxa* implementation. However, figure S11 shows that on an aggregated level, the total sum of extirpations remains relatively the same.

Between including the constraint per taxa or aggregated (*Regional all* versus *Regional per taxa*), there are some differences, with the aggregated constraint resulting in a larger number of global

extirpations. Protection of each taxon may force the model to incur in additional mechanisms to protect biodiversity for a particular taxon, for example, amphibians, which as we saw earlier are the taxon with higher shadow prices of satisfying the biodiversity constraint.

For the same scenario, RCP1.9 with 10% reduction in 2030, the *Global per taxa* implementation is the one with the largest decreases in RW biomass production (9.3%), closely followed by *Regional per taxa* (8.4%) and *Regional all* (2.6%). Again, there are no major differences between *Global per taxa* and *Regional per taxa*. When comparing between aggregating taxa or constraining each taxon separately, constraining taxa separately results in less biomass production in 2030 and more biodiversity protection with respect to the baseline.

Shadow prices vary in magnitude for the different ways of incorporating biodiversity as can be seen in Figure S12. This reflects how the different ways of incorporating biodiversity have an effect on how the model prioritizes biodiversity impacts among taxa and ecoregions and therefore how hard it is to satisfy the constraints. For example, when using *Global per taxa*, amphibians are no longer the taxon with the highest shadow price and when using *Regional all* shadow prices decrease significantly (to 576 (2020 USD)) because now the model can interchange taxon protection to satisfy the constraint.This may be the reason why *Regional all* is the only implementation feasible for a 20% reduction, among the three tested.

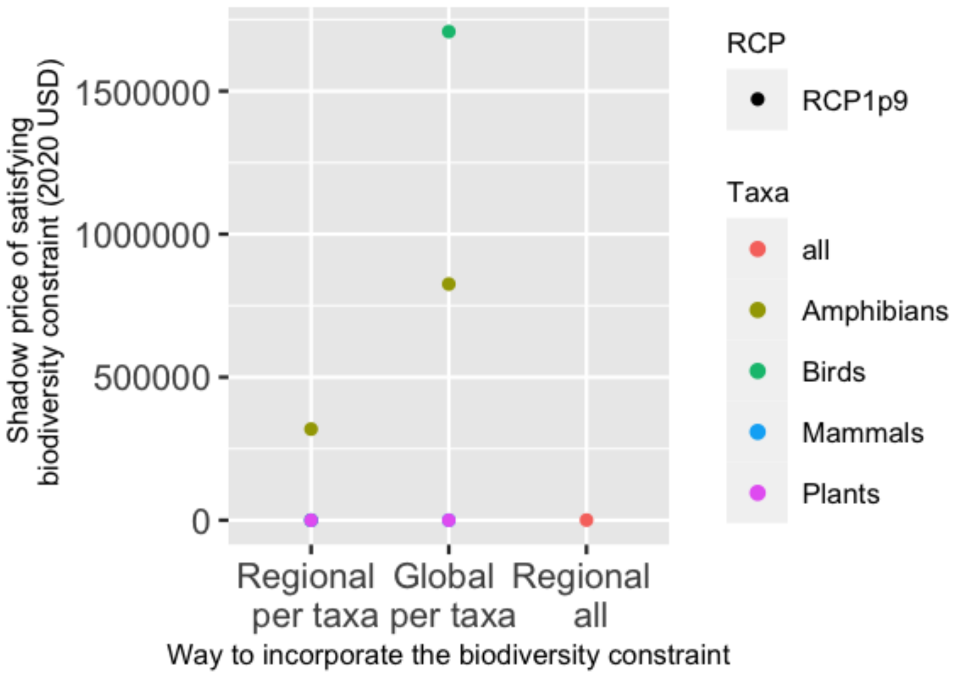


Figure S12: Comparison between shadow prices of the three ways to restrict biodiversity loss, for the RCP1.9 and 10% reduction scenario for 2030. Regional per taxa is used in this study.

# S12 About biodiversity parameters

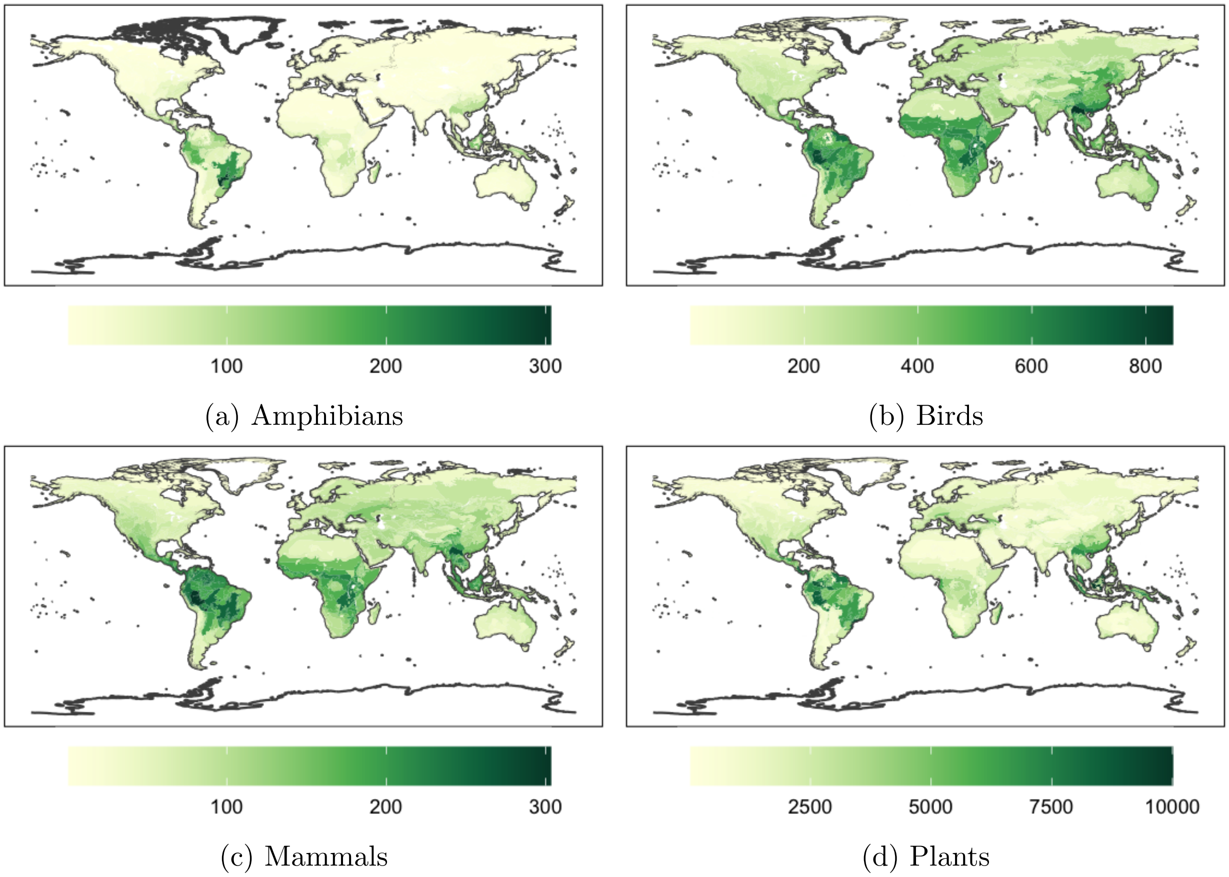


(a) Amphibians (b) Birds

(c) Mammals (d) Plants

Figure S13: Reference species richness $Sorg_{g,l}$ for all ecoregions for each taxon.

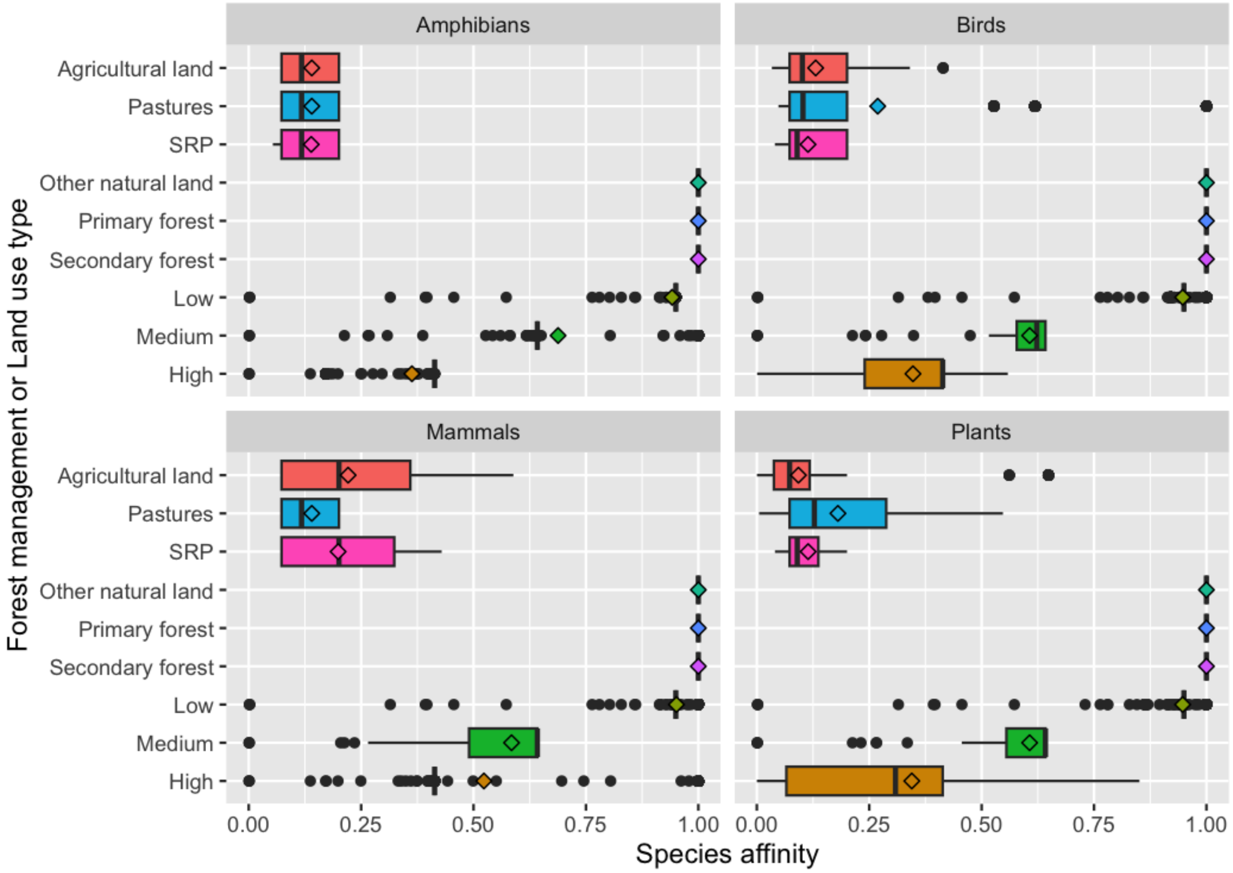


Figure S14: Box plot of affinities $h\_FM_{g,i,l}$ (rows 5-9), $h\_LU_{g,i',l}$ (rows 1,2,4) and $h\_SRP_{g,l}$ (row 3) for all taxon and ecoregions

# References

(1) Havlík, P.; Schneider, U. A.; Schmid, E.; Böttcher, H.; Fritz, S.; Skalský, R.; Aoki, K.; Cara, S. D.; Kindermann, G.; Kraxner, F.; Leduc, S.; McCallum, I.; Mosnier, A.; Sauer, T.; Obersteiner, M. Global land-use implications of first and second generation biofuel targets. *Energy Policy* **2011**, *39*, 5690–5702.

(2) Havlik, P.; Valin, H.; Herrero, M.; Obersteiner, M.; Schmid, E.; Rufino, M. C.; Mosnier, A.; Thornton, P. K.; Bottcher, H.; Conant, R. T.; Frank, S.; Fritz, S.; Fuss, S.; Kraxner, F.; Notenbaert, A. Climate change mitigation through livestock system transitions. *Proc Natl Acad Sci U S A* **2014**, *111*, 3709–14.

(3) Riahi, K. et al. The Shared Socioeconomic Pathways and their energy, land use, and greenhouse gas emissions implications: An overview. *Global Environmental Change* **2017**, *42*, 153–168.

(4) Fricko, O. et al. The marker quantification of the Shared Socioeconomic Pathway 2: A middle-of-the-road scenario for the 21st century. *Global Environmental Change* **2017**, *42*, 251–267.

(5) Rogelj, J. et al. Scenarios towards limiting global mean temperature increase below 1.5 C. *Nature Climate Change* **2018**, *8*, 325–332.

(6) Lauri, P.; Forsell, N.; Gusti, M.; Korosuo, A.; Havlík, P.; Obersteiner, M. Global Woody Biomass Harvest Volumes and Forest Area Use Under Different SSP-RCP Scenarios. *Journal of Forest Economics* **2019**, *34*, 285–309.

(7) Chaudhary, A.; Burivalova, Z.; Koh, L. P.; Hellweg, S. Impact of Forest Management on Species Richness: Global Meta-Analysis and Economic Trade-Offs. *Sci Rep* **2016**, *6*, 23954.

(8) Chaudhary, A.; Verones, F.; de Baan, L.; Hellweg, S. Quantifying Land Use Impacts on Biodiversity: Combining Species-Area Models and Vulnerability Indicators. *Environ Sci Technol* **2015**, *49*, 9987–95.

(9) Kindermann, G. E.; Obersteiner, M.; Rametsteiner, E.; McCallum, I. Predicting the deforestation-trend under different carbon-prices. *Carbon Balance Manag* **2006**, *1*, 15.

(10) Azuero-Pedraza, C. G.; Thomas, V. M. Incorporating biodiversity impacts in land use decisions [In review]. *Ecological Modelling* **2023**,

(11) Humpenöder, F.; Popp, A.; Dietrich, J. P.; Klein, D.; Lotze-Campen, H.; Bonsch, M.; Leon Bodirsky, B.; Weindl, I.; Stevanović, M.; Müller, C. Investigating afforestation and bioenergy CCS as climate change mitigation strategies. *Environmental Research Letters* **2014**, *9*.

(12) Mishra, A.; Humpenöder, F.; Dietrich, J. P.; Bodirsky, B. L.; Sohngen, B.; P. O. Reyer, C.; Lotze-Campen, H.; Popp, A. Estimating global land system impacts of timber plantations using MAgPIE 4.3.5. *Geosci. Model Dev.* **2021**, *14*, 6467–6494.

(13) Besnard, S.; Koirala, S.; Santaro, M.; Weber, U.; Nelson, J.; Güter, J.; Herault, B.; Kassi, J.; N'Guessan, A.; Neigh, C.; Poulter, B.; Zhang, T.; Carvalhais, N. Mapping global forest age from forest inventories, biomass and climate data. *Earth System Science Data* **2021**, *13*, 4881–4896.

(14) Kindermann, G. E.; McCallum, I.; Fritz, S.; Obersteiner, M. *A global forest growing stock, biomass and carbon map based on FAO statistics*; 2008; Vol. 42.

(15) FAO, *Global Forest Resources Assessment 2020 Main report*; Report, 2020.

(16) Chaudhary, A.; Brooks, T. M. Land Use Intensity-Specific Global Characterization Factors to Assess Product Biodiversity Footprints. *Environ Sci Technol* **2018**, *52*, 5094–5104.
